# Large language models as versatile predictive engines for notifiable infectious diseases

**DOI:** 10.1371/journal.pdig.0001527

**Published:** 2026-07-08

**Authors:** Xinsheng Wu, Jinyuan Wu, Zhongwen Wang, Xinyue Feng, Ye Yao, Jason J. Ong, Huachun Zou

**Affiliations:** 1 Shanghai Institute of Infectious Disease and Biosecurity, School of Public Health, Fudan University, Shanghai, China; 2 Center for AIDS Research (CFAR), School of Public Health, Key Laboratory of Public Health Safety, Ministry of Education, Fudan University, Shanghai, China; 3 Melbourne Sexual Health Centre, Alfred Health, Melbourne, Australia; 4 School of Translational Medicine, Faculty of Medicine Nursing and Health Sciences, Monash University, Melbourne, Australia; 5 School of Public Health, Southwest Medical University, Luzhou, China; Liverpool John Moores University - City Campus: Liverpool John Moores University, UNITED KINGDOM OF GREAT BRITAIN AND NORTHERN IRELAND

## Abstract

Accurate forecasting of infectious disease cases and deaths is crucial for public health decision-making. Traditional statistical and machine learning approaches may be challenged by complex temporal patterns and heterogeneous surveillance data. We evaluated large language models (LLMs) for infectious disease forecasting. We collected monthly reported cases and deaths data from China National Notifiable Diseases Surveillance System (NNDSS) from January 2009 to February 2025, and monthly reported cases from the United States NNDSS from 2016 to 2023, covering 79 notifiable infectious diseases and 111 forecasting tasks. We evaluated seven models: four statistical models (ARIMA, TGARCH, EGARCH, ETS), two machine learning models (XGBoost, LSTM), and an LLM-based regression model built on Qwen-2.5-3B and fine-tuned using Low-Rank Adaptation (LoRA). Performance was assessed using mean absolute error (MAE), root mean squared error (RMSE), and mean absolute percentage error (MAPE). We used Friedman tests and Nemenyi post-hoc comparisons versus the LLM, reporting mean-rank differences (ΔR; > 0 indicates better LLM ranking). In pooled analyses, the Friedman test indicated significant heterogeneity across models (χ² = 26.24, *P* < 0.001). Nemenyi comparisons showed positive ΔR for all baselines against the LLM, with significance only in the XGBoost–LLM comparison (ΔR = 0.72, *P* < 0.001). By category, significant positive ΔR for all baselines against the LLM was observed in zoonotic infections (XGBoost: ΔR = 1.42, *P* < 0.001) and intestinal infections (TGARCH: ΔR = 1.28, *P* = 0.012; EGARCH: ΔR = 1.98, *P* < 0.001), whereas blood-borne infections showed LLM inferiority (ETS: ΔR=−2.29, *P* = 0.011; XGBoost ΔR=−2.86, *P* < 0.001). Geographically, positive ΔR was significant in China (XGBoost: ΔR = 0.80, *P* = 0.007), while the U.S. stratum was not significant (χ² = 9.04, *P* = 0.171). By outcome, between-model differences were detectable for case-forecasting (χ² = 20.72, *P* = 0.002; XGBoost: ΔR = 0.73, *P* = 0.005), with no detectable differences for death (χ² = 9.30, *P* = 0.158). In conclusion, LoRA fine-tuned LLM showed performance broadly comparable to statistical and machine learning models, with significant positive ΔR mainly observed in case-forecasting and in zoonotic and intestinal infectious diseases.

## Author summary

Accurate forecasting of infectious disease cases and deaths is essential for timely public health preparedness, resource allocation, and epidemic control. Traditional statistical models and machine-learning methods are widely used for infectious disease forecasting, but their performance may be limited when surveillance time series contain complex temporal patterns, heterogeneous disease dynamics, and sparse observations. Large language models have shown strong ability to learn complex representations, but their value for infectious disease time-series forecasting has not been systematically evaluated. In this study, we assessed a fine-tuned large language model for forecasting notifiable infectious diseases using public surveillance data from China and the United States. Across 111 forecasting tasks covering 79 infectious diseases, the large language model showed competitive performance compared with classical statistical and machine-learning models, especially for the Zoonotic Infectious Diseases and Intestinal Infectious Diseases categories. Our findings suggest that fine-tuned large language models can serve as a promising predictive engine for infectious disease surveillance. This work provides evidence and a methodological foundation for future artificial-intelligence-driven public health forecasting systems.

## Introduction

Accurate forecasting of infectious disease cases and deaths is critical for timely public health interventions and epidemic control [1]. Predictive models can help identify epidemic trends early and inform public health interventions by analysing historical and real-time data. By capturing early signals of disease trends, these models can support earlier forecasting of transmission patterns and assist in implementing timely public health measures. This type of models not only helps to optimise the allocation of medical resources and intervention strategies (such as priority vaccination, regional containment and social distancing policies), but also reduces the socio-economic impact while containing the epidemic [2,3].

In recent years, data-driven forecasting methods have garnered a notable surge in attention [4]. Traditional statistical models, such as the autoregressive integrated moving average (ARIMA) model, threshold generalized autoregressive conditional heteroscedasticity (TGARCH), and the exponential smoothing state-space model (ETS), have been widely used in infectious disease time series prediction [5–8]. However, with the increase in data volume and complexity, the effectiveness of these traditional models is often limited by complex time patterns and data heterogeneity, which may not fully adapt to the nonlinear relationships and dynamic changes in the data [9,10]. For example, ARIMA models have difficulty dealing with non-linear relationships and data heterogeneity [7], while TGARCH and EGARCH models are able to capture volatility but perform poorly in dealing with long-term dependencies [11].

The shortcomings of statistical models in dealing with nonlinear relationships have encouraged the widespread application of machine learning methods to infectious disease forecasting, and more and more researches are applying machine learning techniques to mine propagation patterns in historical data. Algorithms such as XGBoost and long short-term memory (LSTM) networks, with their excellent fitting ability and ability to capture complex patterns, provide powerful tools for modelling nonlinear dynamic processes [11,12]. Using clinical symptoms and biomarkers, Alim *et al.* constructed SARIMA and XGBoost models to predict brucellosis [13]; Peng *et al.* combined daily new COVID-19 cases with Google Trends search data to construct a random forest model to predict the epidemic trend in the next 7 days [14]. These advances in predictive algorithms are making infectious disease forecasting an increasingly important tool for public health prevention and control. Beyond traditional surveillance data, machine-learning models have also been applied to other public-health-related signals. Biri et al. [15] forecast the future popularity of anti-vaccine narratives on Twitter using machine-learning models, illustrating that AI-based approaches can capture temporal patterns in public-health-related phenomena.

Large language models (LLMs), with their powerful natural language processing capabilities and deep learning architectures, can capture complex semantic information and temporal dependencies from large text data, and handle more complex patterns and associations [16]. The core concept of LLMs is to train a large amount of text, learn its language patterns, and generate models with certain reasoning capabilities [17]. Since OpenAI released its first Generative Pretrained Transformer (GPT) based on the Transformer architecture in 2018 [18], LLMs have rapidly made significant progress, with the release of GPT-3 marking a leap forward in the field of natural language processing [19]. In recent years, a number of excellent LLMs have emerged in China, such as DeepSeek and Qwen, which have demonstrated strong capabilities in the field of Chinese language processing [20,21]. These models not only perform excellently in language understanding and generation tasks, but also show great promise for medical applications, especially in various tasks such as cardiovascular disease prevention, infectious disease monitoring, and tumour survival prediction [22–25]. More recently, researchers have begun to adapt LLMs themselves to time-series forecasting by reformulating numerical sequences as language-model inputs. LLMTime encodes time series as strings of numerical digits and treats forecasting as next-token prediction, showing that LLMs can perform zero-shot extrapolation on selected forecasting benchmarks [26]. Time-LLM further reprograms time-series inputs into representations aligned with frozen LLM backbones, suggesting that general-purpose LLMs can be adapted to forecasting while keeping the pretrained language-model backbone intact [27]. However, no prior studies have systematically applied pretrained LLMs to strictly framed time-series forecasting tasks, and the potential of LLMs in this area needs to be further explored.

By comparing the predictive performance of traditional statistical models, machine learning models and an LLM, our study conducts a preliminary, systematic benchmarking evaluation of LLM forecasting performance across 79 infectious diseases with different transmission routes for the first time, aiming to provide a new perspective and evidence-based support for the selection of disease prediction models in the field of public health. In addition, our study applies prospects of LLMs in infectious disease prediction, analyses their advantages and challenges, and offers suggestions for future research.

## Methods

### Disease selection criteria and data collection

**Fig 1:**
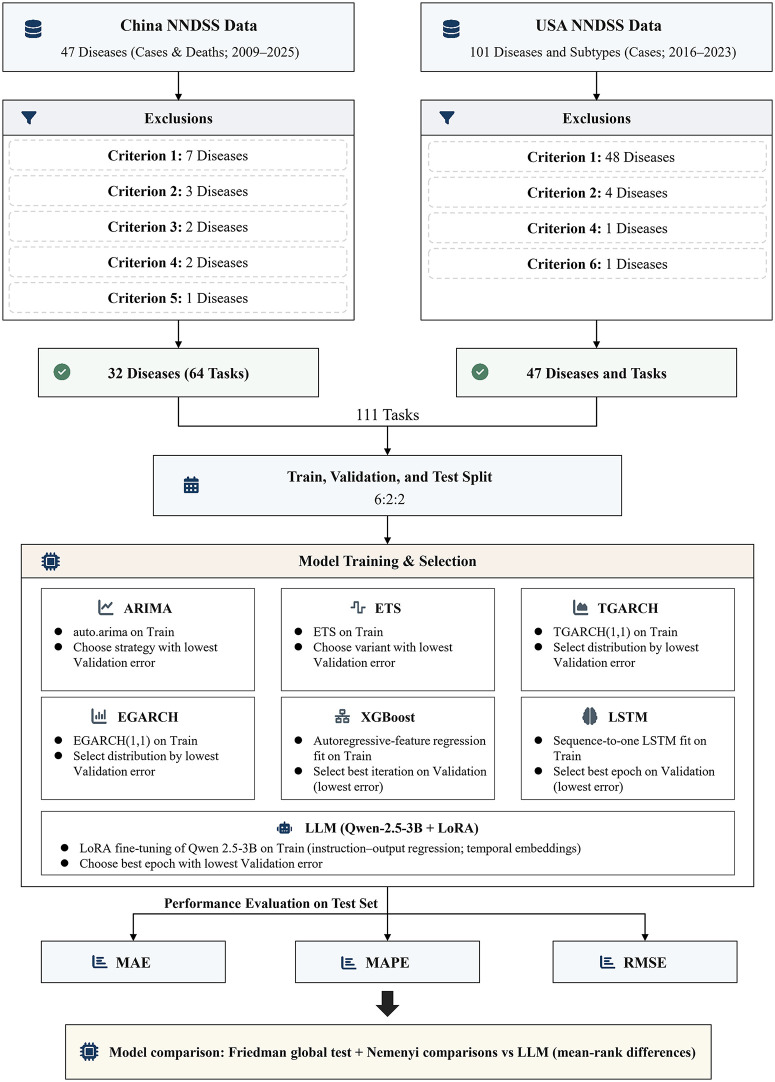
Flow chart. NNDSS, National Notifiable Diseases Surveillance System; ARIMA, autoregressive integrated moving average; TGARCH, threshold generalized autoregressive conditional heteroskedasticity; EGARCH, exponential generalized autoregressive conditional heteroskedasticity; ETS, exponential smoothing state-space model; XGBoost, Extreme Gradient Boosting; LSTM, long short-term memory network; LLM, large language model–based regression; LoRA, low-rank adaptation; MAE, mean absolute error; MAPE, mean absolute percentage error; RMSE, root mean squared error. Criterion 1. Very low reported case counts with long stretches of zeros, leading to unstable estimation. Criterion 2. Recently emerged disease or short surveillance series with insufficient historical data. Criterion 3: Substantial changes in diagnostic or classification practices over time; temporal trends mainly reflect diagnostic rather than epidemiological changes. Criterion 4: Extreme temporal variability with frequent outbreaks or outliers that impair stable parameter estimation and model convergence. Criterion 5: Extremely low endemicity or near-elimination in the study setting. Criterion 6. Animal-only or non-human infection category (e.g., Rabies, Animal), not modeled as a human infectious disease outcome.

We extracted monthly counts of cases and deaths for China’s notifiable infectious diseases (NIDs) from January 2009 to February 2025, and monthly counts of cases for U.S. nationally NIDs from 2016 to 2023. Established in 2004 and continuously upgraded, the National Notifiable Diseases Surveillance System (NNDSS) in China supplied information on 47 diseases (including six hepatitis subtypes) by early 2025. In the United States, the NNDSS is the CDC’s national case-based surveillance system for monitoring the occurrence of nationally notifiable diseases and conditions. It compiles case notifications submitted by U.S. reporting jurisdictions to support situational awareness and public health action (e.g., tracking trends, detecting outbreaks, guiding prevention and control). To ensure robust analysis and model convergence, we applied six prespecified exclusion criteria (Fig 1 and S1–S3 Tables), retaining 32 of 47 NIDs in China and 47 of 101 U.S. NIDs (including available subtypes, where reported). Because the U.S. NNDSS is fundamentally a case-based surveillance system and the publicly available NNDSS dataset we used provides case counts rather than a standardized, routinely available mortality time series across conditions, the U.S. extension was limited to case-only tasks. With this addition, we evaluated model performance across two countries and 111 forecasting tasks in total: 64 China tasks (32 diseases × cases and deaths) and 47 U.S. case tasks. Included diseases were categorized into 6 categories based on their primary modes of transmission: Intestinal Infectious Diseases (IIDs), Blood-borne Diseases (BDs), HIV and Sexually transmitted Diseases (HSTDs), Respiratory Infectious Diseases (RIDs), Zoonotic Infectious Diseases (ZIDs), and Others (S2-S3 Tables). The data source was summarized in S10 Table.

### Model selection

This study compared seven predictive models: four traditional statistical models (ARIMA, TGARCH, EGARCH, and ETS), two machine learning models (XGBoost and LSTM), and one LLM-based regression model. For the traditional statistical models, the ARIMA model was implemented using the “forecast” package in R. The Box–Jenkins methodology was applied, and the optimal model was selected automatically by minimizing the in-sample forecast error. ARIMA orders were chosen by minimizing the corrected AIC (AICc). To enhance robustness, we considered several candidate auto-ARIMA fitting strategies and retained the one achieving the lowest validation mean squared error (MSE). Since ARIMA models have built-in techniques, such as differencing, to handle non-stationarity, no additional transformation was performed on the raw series. The TGARCH and EGARCH models were specified using the “rugarch” package. The TGARCH model was defined with a GARCH(1,1) order and a threshold component to capture asymmetry in the volatility, while the EGARCH model, based on an exponential formulation, was fitted using a hybrid solver. Both TGARCH and EGARCH were fixed at a GARCH(1,1) volatility term and an ARMA(0,0) mean structure. For each model, the conditional distribution was selected from Gaussian and Student-t by minimizing the validation MSE. The ETS model was implemented using the “ets” function from the “forecast” package. This approach employs exponential smoothing techniques to forecast future values by assigning higher weights to more recent observations while capturing trend and seasonality through smoothing parameters. We compared candidate ETS variants (e.g., damped vs non-damped) and selected the specification with the lowest validation MSE.

For the machine learning models, the XGBoost approach was implemented by first constructing lag features from the raw time series. The resulting feature matrix and corresponding target values were then used to train an ensemble of decision trees with the “XGBoost” package, using a square error objective. XGBoost hyperparameters were fixed, and the optimal number of boosting rounds was selected by early stopping on the validation Root Mean Squared Error (RMSE), because RMSE is a monotonic transform of MSE on a fixed validation set, thus yielding the same model-selection ordering while reporting error on the original count scale. No explicit log transformation was applied in the XGBoost pipeline, as the model directly learns from the original lagged features. The LSTM model was developed using the Keras library in R. Prior to training, the series was scaled using a min-max normalization function to facilitate gradient-based optimization, and sequences were generated with a specified lag window. The network architecture consisted of an input layer followed by an LSTM layer with 50 units and a dense output layer producing a single value. Hyperparameters were fixed a priori, and the model was trained for up to 80 epochs with a batch size of 16 using the Adam optimizer. The best epoch was selected by early stopping based on validation loss (MSE), and the inverse normalization was applied to recover predictions on the original scale.

For the LLM-based regression model, we used a pre-trained Qwen-2.5-3B Transformer as its backbone. The Qwen-2.5-3B model, which was pre-trained on an extensive, multilingual, and multimodal dataset (up to 18 trillion tokens), was fine-tuned using LoRA in this study. Qwen-2.5-3B was selected because its large-size vocabulary (≈150 k tokens), trained on massive Chinese corpora, preserves the integrity of our Chinese prompts, and because its moderate scale (3 billion parameters) permits LoRA fine-tuning and inference on a single GPU without resorting to model parallelism, which is an essential practicality for investigators and public-health agencies operating under constrained computational budgets. In the fine-tuning process, only the token embeddings and the final two Transformer layers are unfrozen for training, while the remaining parameters are kept fixed. The LoRA configuration employs a rank of 16 and an alpha scaling factor of 64, with a dropout rate of 0.05. To capture temporal dependencies inherent in the epidemiological data, two additional embedding layers (for year and month) were integrated into the model. The output from the Transformer’s pooled representation was concatenated with these temporal embeddings and passed through a regression head composed of a linear layer with ReLU activation, dropout (at 0.2), and a final linear layer that produces a single output. In the deep learning framework, the outputs were first log-transformed and then scaled using Min-Max normalization, with the scaler fitted on the training data only to ensure consistency. During evaluation, model outputs were inverse-normalized back to the transformed scale and truncated at a minimum of zero before converting them back to the original count scale, ensuring non-negative predictions.

To address concerns that explicit temporal embeddings may advantage the LLM relative to baselines that ingest raw numeric series, we performed a controlled ablation that removed the year and month embedding channels while keeping the remainder of the LLM training and evaluation pipeline unchanged. To evaluate whether the pretrained LLM could have been exposed to and memorized test-period surveillance values during pretraining, we performed a pretraining-contamination sensitivity analysis using a zero-shot baseline. We loaded the frozen base Qwen-2.5-3B model and did not perform any task-specific fine-tuning. For each disease–outcome task, data were sorted chronologically and split into training/validation/test partitions (60%/20%/20%). The pretrained-only model was queried only on the test partition using a constrained prompt that includes only the target month/disease/outcome label and requires a one-line JSON output of a single non-negative integer. To avoid inadvertently parsing numbers contained in the prompt, we decoded generated tokens only and extracted the first valid numeric value using a rule-based parser. We then computed performance on the raw count scale. If test-period values had been memorized during pretraining, this pretrained-only baseline would be expected to return near-correct counts and yield low errors; conversely, poor performance would argue against memorization as the explanation for downstream test results.

### Training and evaluation

In total, 111 tasks were included (Fig 1). After splitting each time series into training, validation, and testing sets (using a 60/20/20 split), the respective model was fitted to the training data. This strict forward split ensures that every prediction is made for time points posterior to all data the model has seen during fitting and hyper-parameter tuning, thereby eliminating look-ahead bias. No rolling or expanding windows were used [28]. In some cases (e.g., XGBoost and LSTM), additional data preparation steps such as scaling or lag feature construction were performed. The forecasting horizon for each task was set equal to the length of the test set, and predictions were generated accordingly. In China, the test set for each task spans November 2021 through February 2025, yielding a fixed 40-month forecasting horizon across all series; in the United States, the test set spans May 2022 through December 2023, yielding a fixed 20-month forecasting horizon across all series. Evaluation metrics, including Mean Absolute Error (MAE), RMSE, and Mean Absolute Percentage Error (MAPE), were computed by comparing the predicted values with the ground truth from the test set. When the true monthly count equalled zero throughout the test horizon, MAPE was undefined; in those cases the denominator-free metrics (MAE and RMSE) were still reported, and MAPE was recorded as “NA”.

For the LLM-based regression model, training was conducted using a mixed-precision strategy on a CUDA-enabled device to accelerate computation and reduce memory requirements. Every monthly observation is encoded as a short Chinese sentence that concatenates calendar information, disease name, and outcome (cases or deaths). For example: “the number of plague cases in January 2009 (in Chinese)”. We adopt this text-serialization primarily as an interface design: it allows a general-purpose pretrained Transformer to consume epidemiological sequences using its native tokenizer while explicitly conditioning on structured metadata without additional feature engineering. In contrast to dedicated time-series foundation models (TSFMs) such as Chronos [29] or TimeGPT [30] that are pretrained to ingest purely numerical sequences, our formulation keeps the input as a unified token sequence in which both the historical counts and the task descriptors are represented in the same space, facilitating disease- and outcome-specific adaptation under small-data fine-tuning (LoRA). Moreover, this text-based interface is naturally extensible to future settings where unstructured context (e.g., brief outbreak reports, policy changes) may be appended alongside the numerical history within the same prompt, which is difficult to express in strictly numeric-only TSFM pipelines. These prompts were tokenised with Qwen-2.5-3B’s native ≈150 k-token vocabulary. The optimizer used was AdamW with a learning rate of 5 × 10 ⁻ ⁵ and weight decay of 0.05; a linear scheduler with up to 200 warm-up steps was applied. Training was run for a maximum of 20 epochs, with early stopping employed if validation performance (measured by MSE) did not improve for 3 consecutive epochs. At each epoch, the training loss was computed with a custom weighting scheme that multiplies the MSE loss for each sample by a factor of (1.0 + α × target), where α was set to 10.0. After training, the best model parameters were saved and used to generate predictions on the test set. Because the model outputs were trained on log-transformed and normalized targets, inverse transformations were applied to recover predictions on the original scale.

### Model interpretability

To improve transparency of the LLM-based forecasting model, we conducted a post-hoc interpretability analysis based on attention attribution [31–33]. For each task-specific fine-tuned model, we extracted the final-layer self-attention matrix during inference and averaged attention across heads. We then computed a token-level attention profile using the attention distribution of the final query token (last-token attention), and normalized attention weights such that they sum to one across the input sequence. We summarized how attention is distributed across three semantically-defined prompt components: (i) Date (year and month), (ii) Disease, and (iii) Outcome (cases vs deaths). Component weights were computed as the sum of normalized token attention weights assigned to each component, yielding a 3-part decomposition per task. We reported component-weight distributions, and we visualized all tasks using an heatmap grouped by disease transmission categories.

### Statistical analyses

Monthly reported case and death counts for 32 NIDs from January 2009 to February 2025 in China and monthly reported cases for 47 NIDs from 2016 to 2023 in the United States were aggregated by disease and transmission category. For each disease, we computed the median and interquartile range (IQR) of monthly cases and deaths, identified the maximum monthly case and death counts, and recorded the dates on which these maxima occurred. To statistically compare predictive performance across models, we used a rank-based non-parametric testing framework. We first applied the Friedman test as a global test for differences among multiple models, and then performed Nemenyi post-hoc comparisons to compare predictive performance between the LLM and each competing model (ARIMA, TGARCH, EGARCH, ETS, XGBoost, and LSTM). The primary comparison was the pooled comparison of model performance across all disease–outcome tasks, followed by Nemenyi-adjusted pairwise comparisons between each baseline model and the LLM. Subgroup analyses (exploratory) by metrics (MAE, RMSE, and MAPE), disease category, country, and outcome (case and death) were treated as secondary exploratory analyses, and interpreted according to the direction and magnitude of ΔR together with *P* values.

To quantify the magnitude and direction of pairwise differences, we report the mean-rank difference:


$$\begin{array}{c} \Delta R={\left(\overset{―}{R}\right)}_{\left\{Comparator\right\}}-{\left(\overset{―}{R}\right)}_{\left\{LLM\right\}} \end{array}$$

where $\bar{R}$ denotes the mean-rank difference across the relevant task units. Under this definition, ΔR>0 indicates that the LLM achieves a better (lower) mean rank than the comparator, whereas ΔR<0 indicates worse performance. Statistical significance was assessed using two-sided Nemenyi-adjusted *P*-values, with *P* < 0.05 considered statistically significant.

To evaluate whether the model comparison was stable across different stages of the test period, we performed a temporal sensitivity analysis using the same framework as the primary analysis. We used January 2023 as a calendar cutoff within the available test period, because 2023 corresponded to major transitions in pandemic response and reporting context, including China’s management of COVID-19 as a Class B infectious disease from January 8, 2023, WHO’s declaration on May 5, 2023 that COVID-19 was no longer a public health emergency of international concern, and the end of the U.S. federal COVID-19 public health emergency on May 11, 2023. Model performance was compared across the full test period, months before 2023, and months from 2023 onward. To further characterize settings in which LLM performance was less favorable, we calculated relative MAE, relative MAPE, and relative RMSE for each disease–outcome task. Each relative error metric was defined as the LLM error metric divided by the lowest corresponding error metric achieved by any baseline model for the same task. Values greater than 1 indicate higher error for the LLM than for the best baseline model. We summarized these relative error metrics by disease category, country, and outcome using medians and interquartile ranges. The data processing and modeling for this study were conducted using Python 3.12 (Python Software Foundation, Wilmington, Delaware, USA) and R 4.2.1 (R Foundation for Statistical Computing, Vienna, Austria).

### Role of the funding source

The funders had no role in the study design, data collection, analysis, interpretation, or report writing.

## Results

### Characteristics of 79 NIDs

S2 Table provided a comprehensive overview of the 32 NIDs in China by transmission category and outcome. In the IIDs, median monthly case counts ranged from 847 (IQR 583–1164) (Typhoid and Paratyphoid Fever) to 89 298 (67898–108357) (Other Infectious Diarrheal Diseases), while death counts remained near zero (maximum of 8 in Bacterial and Amoebic Dysentery in July 2009). Within HSTDs, case medians were highest for Syphilis (40 071, 35152–46951; peak March 2024) and lowest for AIDS (4 466, 3330–5322; peak December 2018), whereas AIDS deaths had a median of 1 232 (962–1657) (peak December 2021) and Syphilis deaths only 6 (4–8) (peak January 2020). Among BDs, Hepatitis B exhibited a median of 100 891 (93289–109930) cases (peak March 2024) and 40 (31–50) deaths (peak December 2009), whereas Hepatitis C showed lower case but a late-year death peak in December 2024. RIDs were dominated by Influenza (median 21 536 cases, 10644–77045, peak December 2023; death median 1, 0–4, peak December 2009) and Tuberculosis (median 92 266 cases, 72885–105745, peak June 2009; death median 169, 142–208, peak July 2009). ZIDs demonstrated generally lower medians—e.g., Dengue Fever cases median 28 (7–193) (peak October 2014), Rabies deaths median 44 (15–95) (peak December 2009)—with variable peaks from 2009–2024. Finally, in the Others category, AHC had a median of 2 633 cases (2068–3324, peak September 2010) and Neonatal Tetanus only 11 (3–51, peak August 2009), with both showing minimal mortality.

S3 Table provided a comprehensive overview of the 47 NIDs in the United States by transmission category. In the IIDs, Campylobacteriosis had the highest median burden (5 232, 4 439–6 528), whereas Salmonella *Paratyphi* cases were rare (3, 0–11); several pathogens peaked in summer, including Cyclosporiasis (maximum 2 102 in July 2023) and STEC (maximum 2 695 in July 2023). In HSTDs, *Chlamydia trachomatis* cases dominated (median 134 431, 125 580–157 954; maximum August 2019), followed by Gonorrhea (median 50 242, 44 520–56 263; maximum October 2020) and Syphilis (median 10 894, 8 773–15 446; maximum April 2023), while HIV diagnoses remained much lower (median 3 024, 2 579–3 242; maximum August 2022). Among BDs, acute hepatitis C exceeded acute hepatitis B in median case (427, 348–495 vs 224, 172–264), and perinatal hepatitis C cases were uncommon (15, 5–20). RIDs showed wide variation, with higher medians for Coccidioidomycosis (1 364, 1 186–1 703) and invasive pneumococcal disease (all ages) (1 330, 876–1 966), but sporadic peaks for low-incidence diseases such as Measles (maximum April 2019) and Mumps (maximum December 2016). ZIDs included pronounced summer peaks for Lyme disease (median 2 414, 1 193–4 590; maximum July 2023) and other tick-borne diseases, while malaria and dengue peaked in late summer 2023. Finally, most “Other” infections remained low-incidence, except *Candida auris* (clinical), which reached its maximum in December 2023.

### Model comparison

**Fig 2:**
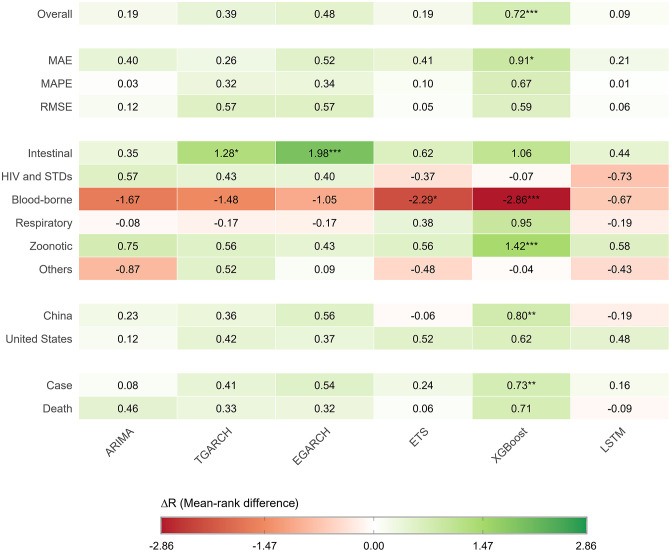
Heatmap of mean rank differences (ΔR) for baseline models versus the LLM. Positive values indicate better performance of the LLM. Stars: * Nemenyi *P* < 0.05, ** *P* < 0.01, *** *P* < 0.001. LLM, large language model–based regression; ARIMA, autoregressive integrated moving average; TGARCH, threshold generalized autoregressive conditional heteroskedasticity; EGARCH, exponential generalized autoregressive conditional heteroskedasticity; ETS, exponential smoothing state-space model; XGBoost, Extreme Gradient Boosting; LSTM, long short-term memory network; MAE, mean absolute error; MAPE, mean absolute percentage error; RMSE, root mean squared error.

In the pooled overall analysis, the Friedman test indicated significant heterogeneity across models (χ² = 26.24, P < 0.001; Tables 1, 2, and S4 Table). In Nemenyi post-hoc comparisons against the LLM, the ΔR (mean-rank difference) were consistently positive for all competing baselines, indicating that the LLM tended to achieve better ranks across tasks. Specifically, the LLM achieved better average ranks than ARIMA (ΔR = 0.19, P = 0.930; Fig 2 and Table 3), TGARCH (ΔR = 0.39, P = 0.253), EGARCH (ΔR = 0.48, P = 0.069), ETS (ΔR = 0.19, P = 0.922), and LSTM (ΔR = 0.09, P = 0.998), with a statistically significant difference after Nemenyi correction specifically in the XGBoost–LLM comparison (ΔR = 0.72, P < 0.001).

**Table 1:** Prediction performance metrics of all models on the test set for case prediction.

Category	Model	MAE (median and IQR)	MAPE (median and IQR)	RMSE (median and IQR)
Intestinal	ARIMA	458.79 (125.69-967.66)	36.97 (34.08-53.44)	581.22 (264.68-1095.66)
Intestinal	EGARCH	425.75 (155.05-874.29)	43.07 (26.10-93.98)	572.89 (208.07-908.31)
Intestinal	ETS	492.68 (113.72-962.28)	39.94 (34.32-74.88)	622.55 (181.13-1090.91)
Intestinal	LLM	385.26 (165.41-841.01)	39.13 (28.60-68.72)	463.68 (300.13-983.82)
Intestinal	LSTM	476.83 (218.94-923.70)	41.34 (34.45-71.57)	558.62 (221.19-1057.04)
Intestinal	TGARCH	404.14 (133.13-865.03)	36.66 (21.33-98.48)	566.11 (187.99-898.78)
Intestinal	XGBoost	641.75 (232.52-981.65)	39.64 (28.95-80.69)	642.52 (291.56-1108.04)
HIV and STDs	ARIMA	4337.76 (2622.56-6255.72)	23.96 (14.13-32.40)	4766.00 (2838.73-7632.15)
HIV and STDs	EGARCH	6164.68 (1006.19-10714.97)	13.36 (9.89-23.58)	8462.73 (1265.75-11860.54)
HIV and STDs	ETS	4031.13 (1990.72-10672.43)	22.19 (17.36-30.47)	4477.76 (2183.39-12439.55)
HIV and STDs	LLM	3418.34 (1415.88-9003.64)	22.50 (14.84-23.86)	4016.18 (1708.00-10335.68)
HIV and STDs	LSTM	5133.79 (1123.26-9477.82)	19.17 (13.78-25.11)	6468.39 (1365.98-10468.06)
HIV and STDs	TGARCH	6145.02 (1011.50-10639.96)	15.91 (12.07-23.22)	8434.57 (1269.96-11608.60)
HIV and STDs	XGBoost	5218.22 (1455.70-7582.61)	20.20 (14.97-26.10)	6141.57 (1761.27-8489.76)
Blood-borne	ARIMA	128.99 (21.73-7432.60)	26.66 (17.52-29.89)	142.14 (29.75-8073.91)
Blood-borne	EGARCH	81.89 (77.15-2345.01)	20.65 (15.75-39.05)	91.60 (86.66-2861.14)
Blood-borne	ETS	71.56 (26.75-2368.84)	16.79 (14.61-18.30)	83.88 (34.76-3119.78)
Blood-borne	LLM	100.83 (75.25-5601.62)	28.70 (22.90-39.65)	116.99 (81.05-6434.88)
Blood-borne	LSTM	47.88 (28.66-2644.41)	16.50 (14.90-20.43)	61.52 (35.90-3508.60)
Blood-borne	TGARCH	109.97 (84.08-2350.24)	22.70 (20.43-34.16)	123.60 (88.74-2863.22)
Blood-borne	XGBoost	43.13 (23.75-2512.90)	13.89 (13.75-19.97)	55.09 (28.72-2962.11)
Respiratory	ARIMA	139.19 (15.37-512.25)	198.78 (20.53-726.94)	61.32 (27.60-113.44)
Respiratory	EGARCH	221.31 (24.60-554.98)	226.48 (27.62-784.36)	60.63 (36.75-133.99)
Respiratory	ETS	200.88 (18.40-638.48)	268.09 (20.61-807.00)	67.33 (50.52-123.50)
Respiratory	LLM	204.35 (14.07-866.40)	268.32 (18.50-1339.44)	54.96 (39.85-86.73)
Respiratory	LSTM	259.89 (13.25-937.92)	322.35 (15.96-1099.87)	50.32 (35.60-101.14)
Respiratory	TGARCH	229.72 (23.05-641.03)	282.64 (26.81-769.59)	73.55 (36.76-146.08)
Respiratory	XGBoost	221.64 (20.29-1460.73)	292.38 (24.05-1558.85)	64.02 (50.99-142.19)
Zoonotic	ARIMA	77.88 (11.29-221.46)	72.91 (40.60-357.78)	102.49 (12.11-295.23)
Zoonotic	EGARCH	75.43 (8.54-223.56)	84.90 (43.95-139.39)	97.74 (10.87-385.50)
Zoonotic	ETS	61.84 (10.23-134.10)	56.96 (46.50-97.07)	86.05 (12.04-193.19)
Zoonotic	LLM	56.78 (10.74-150.79)	67.35 (48.60-116.05)	77.37 (12.76-187.07)
Zoonotic	LSTM	69.82 (11.26-219.31)	79.68 (53.66-210.04)	85.22 (14.78-359.96)
Zoonotic	TGARCH	73.53 (8.55-246.24)	83.81 (48.66-181.90)	95.02 (10.71-362.97)
Zoonotic	XGBoost	94.40 (10.78-321.98)	108.73 (51.45-383.87)	120.60 (12.20-447.06)
Others	ARIMA	4.95 (2.95-119.23)	74.39 (52.66-111.52)	5.40 (3.43-142.55)
Others	EGARCH	18.26 (2.92-153.67)	67.91 (48.19-91.74)	18.65 (3.40-173.99)
Others	ETS	3.29 (2.90-124.04)	68.63 (52.46-76.77)	3.93 (3.30-147.65)
Others	LLM	6.42 (2.66-131.86)	74.48 (61.19-121.69)	7.35 (3.30-154.39)
Others	LSTM	4.15 (2.87-173.75)	87.95 (50.17-125.51)	4.65 (3.65-191.46)
Others	TGARCH	5.80 (2.94-147.56)	71.73 (48.73-92.71)	6.22 (3.40-167.88)
Others	XGBoost	5.09 (2.89-147.32)	84.32 (68.28-111.35)	5.53 (3.38-167.63)

MAE: Mean Absolute Error; MAPE: Mean Absolute Percentage Error; RMSE: Root Mean Squared Error. Each category’s included diseases were listed in S2–S3 Tables.

**Table 2:** Prediction performance metrics of all models on the test set for death prediction.

Category	Model	MAE (median and IQR)	MAPE (median and IQR)	RMSE (median and IQR)
Intestinal	ARIMA	0.45 (0.38-1.11)	65.93 (64.32-68.97)	0.49 (0.39-1.26)
Intestinal	EGARCH	0.69 (0.66-1.16)	56.91 (38.55-72.85)	0.70 (0.67-1.33)
Intestinal	ETS	0.46 (0.35-1.11)	70.31 (63.30-75.18)	0.50 (0.37-1.26)
Intestinal	LLM	0.83 (0.38-1.03)	59.88 (51.63-74.90)	0.86 (0.63-1.19)
Intestinal	LSTM	0.35 (0.34-0.83)	70.85 (33.19-75.23)	0.43 (0.37-0.92)
Intestinal	TGARCH	0.67 (0.61-1.17)	57.29 (40.91-71.47)	0.68 (0.62-1.34)
Intestinal	XGBoost	0.56 (0.47-1.17)	57.71 (54.64-67.27)	0.58 (0.47-1.35)
HIV and STDs	ARIMA	3.54 (1.88-224.92)	86.10 (56.53-126.87)	3.94 (2.14-251.10)
HIV and STDs	EGARCH	3.52 (1.88-370.61)	85.39 (63.35-126.10)	3.92 (2.13-390.93)
HIV and STDs	ETS	3.02 (1.62-97.64)	86.21 (48.30-115.48)	3.42 (1.88-133.52)
HIV and STDs	LLM	2.63 (1.44-209.77)	93.25 (58.52-106.23)	3.12 (1.75-238.54)
HIV and STDs	LSTM	2.35 (1.30-284.93)	82.85 (57.08-97.28)	2.73 (1.53-310.82)
HIV and STDs	TGARCH	3.45 (1.86-374.17)	80.29 (61.01-122.03)	3.85 (2.09-394.31)
HIV and STDs	XGBoost	3.49 (1.85-354.76)	89.48 (64.43-127.47)	3.89 (2.11-377.94)
Blood-borne	ARIMA	68.35 (39.71-96.99)	58.25 (52.90-63.60)	102.82 (57.80-147.84)
Blood-borne	EGARCH	68.38 (39.77-96.99)	58.42 (53.18-63.66)	102.86 (57.87-147.84)
Blood-borne	ETS	69.90 (42.19-97.61)	64.93 (63.14-66.73)	104.45 (60.36-148.53)
Blood-borne	LLM	73.32 (46.32-100.32)	74.74 (73.17-76.30)	107.68 (64.32-151.04)
Blood-borne	LSTM	71.13 (43.10-99.16)	68.23 (65.92-70.54)	105.40 (61.11-149.68)
Blood-borne	TGARCH	68.11 (39.63-96.59)	58.03 (52.95-63.10)	102.64 (57.75-147.52)
Blood-borne	XGBoost	69.74 (42.02-97.47)	64.49 (62.56-66.41)	104.30 (60.21-148.40)
Respiratory	ARIMA	0.70 (0.10-3.70)	90.85 (44.51-95.69)	0.75 (0.17-3.90)
Respiratory	EGARCH	0.39 (0.06-2.17)	95.93 (46.38-96.09)	0.39 (0.16-2.85)
Respiratory	ETS	0.58 (0.06-1.71)	47.92 (46.83-95.80)	0.65 (0.16-2.95)
Respiratory	LLM	0.28 (0.03-2.54)	100.00 (61.14-100.00)	0.34 (0.16-3.82)
Respiratory	LSTM	0.31 (0.05-3.20)	96.91 (47.20-97.22)	0.31 (0.16-3.40)
Respiratory	TGARCH	0.40 (0.04-1.64)	67.30 (47.35-97.21)	0.40 (0.16-2.89)
Respiratory	XGBoost	0.44 (0.10-7.92)	90.58 (59.17-95.69)	0.44 (0.17-9.41)
Zoonotic	ARIMA	0.32 (0.14-1.58)	93.98 (82.21-170.74)	0.48 (0.30-1.98)
Zoonotic	EGARCH	0.28 (0.11-1.10)	91.18 (83.15-99.78)	0.49 (0.30-1.22)
Zoonotic	ETS	0.38 (0.15-1.88)	84.41 (65.29-95.87)	0.53 (0.30-2.52)
Zoonotic	LLM	0.29 (0.10-1.08)	94.62 (76.08-99.28)	0.47 (0.28-1.34)
Zoonotic	LSTM	0.33 (0.14-1.34)	92.26 (83.44-149.94)	0.49 (0.30-1.52)
Zoonotic	TGARCH	0.24 (0.07-1.64)	99.74 (96.57-190.25)	0.51 (0.30-1.75)
Zoonotic	XGBoost	0.34 (0.14-1.75)	94.84 (81.34-185.90)	0.49 (0.30-2.11)
Others	ARIMA	0.04 (0.02-0.06)	86.13 (86.13-86.13)	0.08 (0.04-0.12)
Others	EGARCH	0.36 (0.18-0.54)	26.55 (26.55-26.55)	0.36 (0.18-0.54)
Others	ETS	0.29 (0.15-0.44)	148.64 (148.64-148.64)	0.31 (0.16-0.45)
Others	LLM	0.16 (0.08-0.24)	36.28 (36.28-36.28)	0.20 (0.11-0.29)
Others	LSTM	0.28 (0.15-0.42)	44.55 (44.55-44.55)	0.28 (0.15-0.42)
Others	TGARCH	0.37 (0.19-0.56)	23.80 (23.80-23.80)	0.38 (0.19-0.56)
Others	XGBoost	0.15 (0.08-0.23)	71.15 (71.15-71.15)	0.16 (0.08-0.23)

MAE: Mean Absolute Error; MAPE: Mean Absolute Percentage Error; RMSE: Root Mean Squared Error. Each category’s included diseases were listed in S2–S3 Tables.

**Table 3:** Post-hoc comparisons for baseline models versus the LLM using mean rank differences (ΔR).

Stratum	ARIMA		TGARCH		EGARCH		ETS		XGBoost		LSTM	
	ΔR	*P* value	ΔR	*P* value	ΔR	*P* value	ΔR	*P* value	ΔR	*P* value	ΔR	*P* value
Overall	0.19	0.930	0.39	0.253	0.48	0.069	0.19	0.922	0.72	<0.001	0.09	0.998
MAE	0.40	0.811	0.26	0.973	0.52	0.557	0.41	0.786	0.91	0.028	0.21	0.992
MAPE	0.03	1.000	0.32	0.932	0.34	0.912	0.10	1.000	0.67	0.276	0.01	1.000
RMSE	0.12	1.000	0.57	0.442	0.57	0.442	0.05	1.000	0.59	0.402	0.06	1.000
Intestinal	0.35	0.968	1.28	0.012	1.98	<0.001	0.62	0.648	1.06	0.072	0.44	0.906
HIV and STDs	0.57	0.951	0.43	0.987	0.40	0.992	-0.37	0.995	-0.07	1.000	-0.73	0.845
Blood-borne	-1.67	0.159	-1.48	0.288	-1.05	0.701	-2.29	0.011	-2.86	<0.001	-0.67	0.954
Respiratory	-0.08	1.000	-0.17	0.999	-0.17	0.999	0.38	0.925	0.95	0.088	-0.19	0.998
Zoonotic	0.75	0.135	0.56	0.472	0.43	0.761	0.56	0.472	1.42	<0.001	0.58	0.431
Others	-0.87	0.820	0.52	0.983	0.09	1.000	-0.48	0.989	-0.04	1.000	-0.43	0.994
China	0.23	0.947	0.36	0.677	0.56	0.152	-0.06	1.000	0.80	0.007	-0.19	0.978
United States	0.12	0.999	0.42	0.665	0.37	0.791	0.52	0.389	0.62	0.188	0.48	0.516
Case	0.08	1.000	0.41	0.388	0.54	0.095	0.24	0.890	0.73	0.005	0.16	0.982
Death	0.46	0.794	0.33	0.946	0.32	0.954	0.06	1.000	0.71	0.291	-0.09	1.000

ΔR, mean-rank difference. LLM, large language model–based regression; ARIMA, autoregressive integrated moving average; TGARCH, threshold generalized autoregressive conditional heteroskedasticity; EGARCH, exponential generalized autoregressive conditional heteroskedasticity; ETS, exponential smoothing state-space model; XGBoost, Extreme Gradient Boosting; LSTM, long short-term memory network; MAE, mean absolute error; MAPE, mean absolute percentage error; RMSE, root mean squared error.

### Subgroup analyses (exploratory)

When stratified by disease category, significant positive ΔR was mainly observed in zoonotic and intestinal infection comparisons. In zoonotic diseases, the global Friedman test remained significant (χ² = 25.48, P < 0.001; S4 Table), and the XGBoost–LLM comparison showed significant positive ΔR (ΔR = 1.42, P < 0.001; Fig 2 and Table 3) alongside positive ΔR versus all other comparators (e.g., ARIMA ΔR = 0.75, P = 0.135; TGARCH ΔR = 0.56, P = 0.472; EGARCH ΔR = 0.43, P = 0.761; ETS ΔR = 0.56, P = 0.472; LSTM ΔR = 0.58, P = 0.431; all Nemenyi P > 0.05 except XGBoost). In intestinal diseases, the global Friedman test was also significant (χ² = 38.03, P < 0.001), and the TGARCH–LLM (ΔR = 1.28, P = 0.012) and EGARCH–LLM comparisons (ΔR = 1.98, P < 0.001) showed significant positive ΔR, while still showing positive but non-significant ΔR versus ARIMA (ΔR = 0.35, P = 0.968), ETS (ΔR = 0.62, P = 0.648), XGBoost (ΔR = 1.06, P = 0.072), and LSTM (ΔR = 0.44, P = 0.906).

In contrast, blood-borne infections represented the clearest category where the LLM did not dominate: the global Friedman test was significant (χ² = 25.20, P < 0.001), but the LLM had negative ΔR versus ETS (ΔR=−2.29, P = 0.011) and XGBoost (ΔR=−2.86, P < 0.001), indicating worse mean ranks than these comparators in this subgroup. For respiratory infections, although the global Friedman test suggested heterogeneity (χ² = 17.99, P = 0.006), differences versus the LLM were mixed in direction and not statistically significant. For HIV and STDs and the residual “Others” category, neither the global test nor the comparisons between models provided evidence of a consistent LLM advantage (all Nemenyi P > 0.05; directions varied).

Geographically, the China stratum showed significant global heterogeneity across models (χ² = 30.57, P < 0.001), with significant positive ΔR only in the XGBoost–LLM comparison (ΔR = 0.80, P = 0.007), whereas in the United States stratum the global test was not significant (χ² = 9.04, P = 0.171) and no Nemenyi-adjusted comparison reached P < 0.05 despite mixed (but mostly positive) ΔR against several models. By outcome type, between-model differences were detectable for case forecasting, where the global Friedman test was significant (χ² = 20.72, P = 0.002) and the XGBoost–LLM comparison showed significant positive ΔR (ΔR = 0.73, P = 0.005). For death forecasting, the global test was not significant (χ² = 9.30, P = 0.158) and comparisons between models showed no evidence of statistically detectable differences.

The temporal sensitivity analysis showed consistent results across evaluation windows (S9 Table; S2 Fig). The Friedman test remained significant in the full test period (*P* < 0.001), months before 2023 (*P* < 0.001), and months from 2023 onward (*P* = 0.003). Across all three windows, ΔR values were positive for all baseline models versus the LLM. In the diagnostic analysis, error ratio varied by disease category, country, and outcome (S2 Fig). BDs had the highest median relative errors across all three metrics, with a relative MAE of 1.97 (1.38–2.36), a relative MAPE of 1.72 (1.34–2.29), and a relative RMSE of 1.76 (1.32–2.19). Relative errors were broadly similar between China and the United States and across outcomes.

### Model interpretability

**Fig 3:**
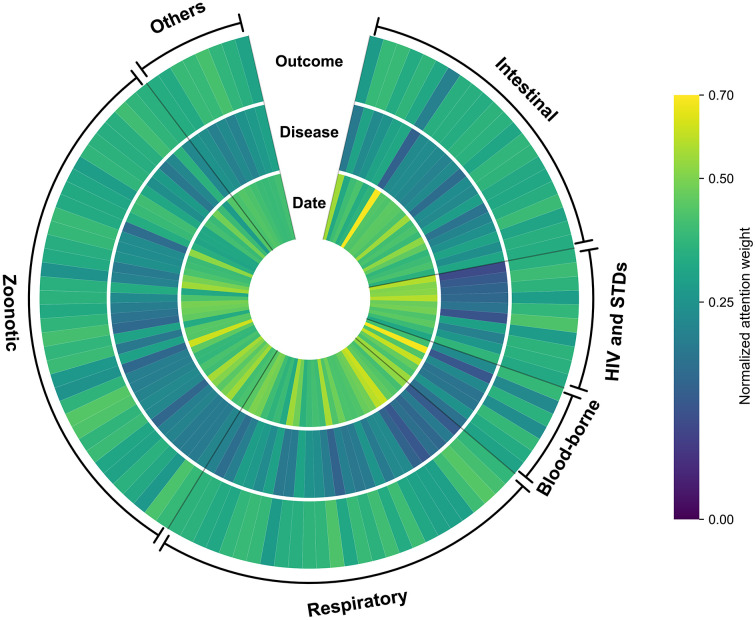
Attention-based component attribution across all disease–outcome forecasting tasks, grouped by transmission category. Each sector denotes one disease–outcome task; inner-to-outer rings correspond to Date, Disease, and Outcome component weights (normalized to sum to one per task).

Fig 3 and S5 Table summarized component-level attention attribution across all tasks. Across transmission categories, the model consistently allocated the largest share of attention to Date, indicating strong reliance on temporal context, with median Date attention ranging from 40.3% to 47.9%. The Outcome component was comparatively stable across categories (median 33.1%–35.9%), whereas Disease attention showed greater between-category variability (median 14.5%–24.2%). Notably, Date attention was highest for HSTDs (median 47.9% [43.2–51.5]) and Blood-borne infections (46.3% [42.4–57.5]), while Disease attention was relatively higher for Intestinal infections (22.6% [20.9–27.9]) and Others (24.2% [21.6–25.5]).

### Ablation without temporal embeddings

In the ablation setting (removing temporal embeddings), the overall conclusions were largely unchanged; however, we observed a small number of significance reversals. At the global level, all strata retained the same significance status as in the main analysis (S6 Table). In Nemenyi post-hoc comparisons against the LLM, four contrasts changed significance status: (i) for MAE, the LLM’s advantage over XGBoost was significant in the main analysis (ΔR = 0.91, *P* = 0.028) but became non-significant in the sensitivity analysis (ΔR = 0.80, *P* = 0.083; S1 Fig and S7 Table); (ii) in intestinal infections, the LLM’s advantage over TGARCH was significant in the main analysis (ΔR = 1.28, *P* = 0.012) but became non-significant in the sensitivity analysis (ΔR = 0.98, *P* = 0.126); (iii) in blood-borne infections, inferiority of the LLM versus ETS was significant in the main analysis (ΔR=−2.29, *P* = 0.011) but became non-significant in the sensitivity analysis (ΔR=−1.86, *P* = 0.078); and (iv) in China, the LLM’s advantage over XGBoost was significant in the main analysis (ΔR = 0.80, *P* = 0.007) but became non-significant in the sensitivity analysis (ΔR = 0.51, *P* = 0.254). All other model comparisons retained the same significance status as in the main analysis.

### Pretraining-contamination sensitivity analysis

The zero-shot baseline exhibited extremely poor performance with severe outliers. When pooling all countries and tasks, the median (IQR) was MAE 1.13 × 10^6 (1.70 × 10^5–5.05 × 10^17), MAPE 9.15 × 10^14 (6.41 × 10^5–1.22 × 10^18), and RMSE 3.36 × 10^6 (1.84 × 10^5–3.20 × 10^18). Details were shown in the S8 Table. In contrast, the fine-tuned LLM achieved substantially smaller and epidemiologically plausible errors across disease categories in both case and death prediction (Tables 1 and 2), supporting that predictive performance arises from learning in the supervised time-split training procedure rather than memorization of test-period surveillance counts.

## Discussion

Our study provides the first benchmarking evaluation of LLMs in infectious diseases forecasting tasks. The LLM showed broadly favorable mean ranks (mainly in case-forecasting tasks) but a statistically significant positive ΔR was observed only in the XGBoost–LLM comparison (ΔR = 0.72, *P* < 0.001), while differences versus ARIMA, TGARCH, EGARCH, ETS, and LSTM were not significant. This pattern suggests that, across a diverse set of diseases and outcomes, the LLM is generally competitive and often comparable to strong classical baselines. In subgroup analyses (exploratory), significant positive ΔR was mainly observed in intestinal infections (TGARCH: ΔR = 1.28; EGARCH: ΔR = 1.98) and zoonotic infections (XGBoost: ΔR = 1.42), while performance was mixed and largely non-significant in respiratory infections, HIV and STDs, and the “Others” category. In contrast, blood-borne infections represented the clearest setting where the LLM did not dominate, showing significant negative ΔR versus ETS and XGBoost (ΔR=−2.29 and −2.86, respectively). Finally, outcome-stratified results were driven mainly by case forecasting (global *P* = 0.002), with no statistically detectable differences among models for death forecasting (global *P* = 0.158).

Different transmission mechanisms influence the choice of the optimal forecasting model. LLMs showed a clear comparative advantage mainly in intestinal and zoonotic infections. One possible explanation is that self-attention can capture long-range dependencies and complex non-linear patterns in time series data [34]. By feeding the data one time point after another, the model naturally uses its understanding of context to spot both quick changes and overall trends. This lets it pick up on things like delayed reactions, historical baseline levels, or the impact of public health measures—patterns that are hard to hard‑code into traditional models [35,36]. In addition, the large parameter scale of LLMs such as Qwen and GPT enhances their representation learning capabilities by exploiting extensive prior knowledge acquired during pre-training. By virtue of their billions of parameters, models such as Qwen-2.5-3B and GPT encode extensive linguistic and domain knowledge from pre-training on massive, multilingual text collections, ranging from web pages and news articles to scientific papers and code repositories. This breadth of exposure allows them to internalize complex language patterns, factual information and specialist terminology (including epidemiological concepts), which in turn supports more accurate downstream forecasting of infectious-disease trends [37]. Recent methodological work on transformer-based representation learning further suggests that pre-training can improve predictive performance in sparse and irregular time series, including mortality prediction when labelled data are limited [38]. Similarly, time-series foundation-model studies emphasise that addressing data scarcity and the limitations of small training datasets via large-scale pretraining and synthetic data can enhance generalisation [29]. Therefore, our observation is consistent with the broader methodological literature on data-efficient transformer learning in sparse settings, but this interpretation remains hypothesis-generating and warrants future targeted validation (e.g., explicit analyses of zero-heavy regimes and ablations focused on sparsity) [39,40]. Importantly, the fine-tuned LLM’s test-set performance is in the expected range for time-series forecasting, whereas the pretrained-only recall baseline yields errors that are orders of magnitude larger, consistent with lack of direct memorization of the test-period counts.

However, traditional statistical and machine learning models still have a place for certain diseases. It is worth noting that for blood-borne infections prediction, all traditional statistical models and machine learning models, especially for ETS and XGBoost (statistically significant), have negative mean-rank difference. Although LLM performed well in many tasks, the complementarity between LLM and traditional statistical and machine learning models suggests the potential of integrated modelling strategies and suggests that in the future, fusion strategies of LLM + other models can be explored to improve overall prediction performance, such as using LLM to capture complex trends and using lightweight models for correction or short-term correction [41,42].

Recent TSFMs, such as Chronos [29] and TimeGPT [30], are explicitly pretrained on large corpora of numerical time series and have reported strong zero-shot forecasting performance across diverse domains. Chronos, in particular, tokenizes time series values via scaling and quantization into a fixed vocabulary and trains Transformer language-model architectures on these tokenized sequences, whereas TimeGPT is a dedicated generative pretrained model for time series. Our work differs in goal and interface: we evaluate whether a multilingual general-purpose LLM can be adapted for surveillance forecasting through lightweight task-specific fine-tuning, while using natural-language templates to jointly encode the numeric history and epidemiological descriptors (disease, outcome, and date). We view TSFMs and LLM-based text-serialization as complementary directions. A head-to-head benchmark against representative TSFMs on infectious-disease surveillance series (including settings with sparse counts and heterogeneous transmission mechanisms) is an important next step for future work.

To our knowledge, no prior studies have systematically applied fine-tuned LLMs to strictly framed time-series forecasting tasks; this work thus fills an important methodological gap. Our study highlights several advantages of LLMs in the field of infectious disease prediction. First, LLMs showed broadly competitive performance across a wide range of prediction tasks, with clearer comparative advantages in intestinal and zoonotic infections but weaker performance in blood-borne infections. For each disease, we trained a separate LLM-based model to fully exploit the inherent advantages of the architecture, such as the ability to capture complex temporal patterns and dynamic relationships, without the need for extensive task-specific feature engineering. This is in stark contrast to traditional statistical and machine learning models, which typically require customised design and manual feature selection for each disease type [43]. Second, the LLM can learn temporal dependencies and nonlinear relationships directly from text-serialized historical counts with simple normalization, reducing the need for extensive domain expertise and labour-intensive data pre-processing in the model development process. Finally, LLM has promising potential for future enhancements, as its ability to integrate real-time data streams and cross-modal unstructured information, such as clinical case data, genomic, environmental and social media data, may enable the development of more responsive and comprehensive disease-prediction systems.

Several limitations of this study should be acknowledged. First, although the dataset used covers 79 nationally notifiable infectious diseases in China and the United States, providing a rare combination of completeness, long time span and public availability, it does not fully capture the wider range of global epidemiological contexts. Most countries do not provide openly accessible infectious disease surveillance data, which limited our ability to include more diverse datasets. As a result, while the findings are promising, broader validation using larger and more heterogeneous datasets is needed to confirm the generalisability of the results. The U.S. data was limited to case-only tasks, and the U.S. stratum did not show statistically detectable global differences across models. Therefore, geographical generalisability should be interpreted cautiously. Second, similar to traditional machine learning models, LLMs are inherently “black box” in nature. However, we provided post-hoc attention attribution to offer a coarse-grained interpretation of what the model attends to, while acknowledging that this does not fully explain the decision process. The attention analysis should therefore be viewed as a post-hoc description rather than as a sufficient basis for public-health decision making. Third, large LLMs typically have high computational requirements and high training and deployment costs, which can make it difficult to run them quickly and cost‑effectively in real‑time scenarios. However, our study found that relatively small models can achieve strong predictive performance. Our model has only 3 billion parameters and was successfully trained and deployed on a server equipped with an 80GB NVIDIA A100 Tensor Core GPU, demonstrating the feasibility of using lightweight LLMs for infectious disease prediction. A single disease–outcome task fine-tuning uses about 44.6 GB of GPU memory and completes in roughly 6 minutes (about 11 hours in total). This greatly reduces the computational burden and shows that models with fewer parameters can still provide powerful predictive capabilities, making them easier to use in real-world applications, especially in resource-constrained environments. However, disease–outcome-specific fine-tuning may limit scalability when frequent updating across many diseases is required. Fourth, before widespread implementation, issues such as data privacy and model bias need to be carefully considered [44], and integration of LLM into existing surveillance and early warning systems requires strong interoperability standards and careful system design to ensure reliability and credibility. Additionally, we did not explicitly model the COVID-19 pandemic as a structural break in the surveillance series [45]. The years 2020–2022, during which non-pharmaceutical interventions substantially altered transmission patterns for many infections, were treated as part of the observed historical data, without dedicated change-point terms or intervention indicators. Therefore, results spanning pandemic-era disruptions should be interpreted as fixed-split benchmarking under observed surveillance conditions rather than as explicit estimates of model performance under separate epidemiological regimes. Moreover, the evaluation used a single prespecified 60%–20%–20% forward split. Although this design avoided look-ahead bias and provided a consistent comparison across 111 heterogeneous forecasting tasks, the estimates are conditional on the chosen temporal split and do not fully demonstrate temporal generalization. Finally, the comparison was not fully symmetric. The LLM used text-serialized inputs, structured prompts, temporal embeddings and disease–outcome-specific LoRA fine-tuning, whereas several comparator models used numerical time-series inputs and more constrained parameterizations. The temporal-embedding ablation addressed one aspect of this concern but did not eliminate all differences in model flexibility. Therefore, our findings should be interpreted as evidence of competitiveness under this fine-tuning framework rather than as evidence of universal model superiority.

Based on the current work, future research should focus on several key areas. First, compared to notification-based passive surveillance, which relies on the number of cases, traditional statistical models and machine learning-based predictive models can integrate outpatient records [46], laboratory test results [13,47], social media dynamics [14] and meteorological environmental data [48,49]. Extending LLM-based forecasting to multi-source, heterogeneous inputs (environmental, demographic, behavioural, and genomic) may further improve accuracy and generalisability across settings. Second, deeper integration of LLM with mechanistic epidemiological models can enable these models not only to learn from data, but also to integrate biology and transmission dynamics, resulting in more robust and interpretable predictions. Third, improving the interpretability of LLM-based predictions is critical to increasing confidence and informing public health decisions. Technologies such as attention visualisation, attribution methods or post-hoc interpretability frameworks can be explored [50,51]. Finally, the combination of user interface and real-time data updates will allow for dynamic adjustment of predictions based on constantly changing information, further enhancing the practicality of these models.

In summary, our study provides the first benchmarking evaluation of LLMs in infectious diseases forecasting tasks. Although challenges remain, the research findings highlight the substantial potential of LLMs to improve the accuracy and reliability of infectious disease prediction (mainly in case-forecasting tasks). These results lay the foundation for future innovation in public health forecasting systems driven by artificial intelligence. In the future, a real-time updated and user-friendly infectious disease prediction system based on multi-source unstructured text is warranted.

## Code sharing statement

The code used for statistical analysis in this study is available at XW’s GitHub repository: https://github.com/XS-Wu/LLM_predict_NIDs

## Supporting information

S1 Fig Heatmap of mean rank differences (ΔR) for baseline models versus the LLM (ablation without temporal embeddings). Positive values indicate better performance of the LLM. Stars: * Nemenyi *P* < 0.05, ** *P* < 0.01, *** *P* < 0.001. LLM, large language model–based regression; ARIMA, autoregressive integrated moving average; TGARCH, threshold generalized autoregressive conditional heteroskedasticity; EGARCH, exponential generalized autoregressive conditional heteroskedasticity; ETS, exponential smoothing state-space model; XGBoost, Extreme Gradient Boosting; LSTM, long short-term memory network; MAE, mean absolute error; MAPE, mean absolute percentage error; RMSE, root mean squared error.
(PDF)

S2 Fig Sensitivity and diagnostic analyses of model performance. (A) Mean-rank differences (ΔR) for baseline models versus the LLM across the full test period, months before 2023, and months from 2023 onward. Positive values indicate better performance of the LLM. *P* values are from Friedman tests using the same framework as the primary analysis. (B) Relative MAE, relative MAPE, and relative RMSE for the LLM compared with the best baseline model by disease category, country, and outcome. Each relative error metric was calculated as the LLM error metric divided by the lowest corresponding error metric achieved by any baseline model for the same task. Points show medians, horizontal lines show interquartile ranges, and colors indicate MAE, MAPE, and RMSE. LLM, large language model–based regression; ARIMA, autoregressive integrated moving average; TGARCH, threshold generalized autoregressive conditional heteroskedasticity; EGARCH, exponential generalized autoregressive conditional heteroskedasticity; ETS, exponential smoothing state-space model; XGBoost, Extreme Gradient Boosting; LSTM, long short-term memory network; MAE, mean absolute error; MAPE, mean absolute percentage error; RMSE, root mean squared error.
(PDF)

S1 Table Excluded notifiable infectious diseases and corresponding exclusion reasons. (DOCX)

S2 Table Case and death numbers for 32 notifiable infectious diseases in China from January 2009 to February 2025. (DOCX)

S3 Table Case numbers for 47 notifiable infectious diseases in the United States from 2016 to 2023. (DOCX)

S4 Table Global tests comparing model performance ranks. (DOCX)

S5 Table Component-level attention weights by transmission category. (DOCX)

S6 Table Global tests comparing model performance ranks (ablation without temporal embeddings). (DOCX)

S7 Table Post-hoc comparisons for baseline models versus the LLM using mean rank differences (ΔR) (ablation without temporal embeddings). (DOCX)

S8 Table Zero-shot prediction performance on the test set (pretraining-contamination sensitivity analysis). (DOCX)

S9 Table Sensitivity analysis by evaluation period using mean rank differences (ΔR). (DOCX)

S10 Table Data sources used in the study. (DOCX)
