# Supplementary material for: Large language models as versatile predictive engines for notifiable infectious diseases
Source: PLOS Digit Health. 2026 Jul 8;5(7):e0001527. doi: 10.1371/journal.pdig.0001527 (PMC13345230; doi:10.1371/journal.pdig.0001527)
Supplement: S1 Fig — Positive values indicate better performance of the LLM. Stars: * Nemenyi P < 0.05, ** P < 0.01, *** P < 0.001. LLM, large language model–based regression; ARIMA, autoregressive integrated moving average; TGARCH, threshold generalized autoregressive conditional heteroskedasticity; EGARCH, exponential generalized autoregressive conditional heteroskedasticity; ETS, exponential smoothing state-space model; XGBoost, Extreme Gradient Boosting; LSTM, long short-term memory network; MAE, mean absolute error; MAPE, mean absolute percentage error; RMSE, root mean squared error. (PDF) [file pdig.0001527.s001.pdf]

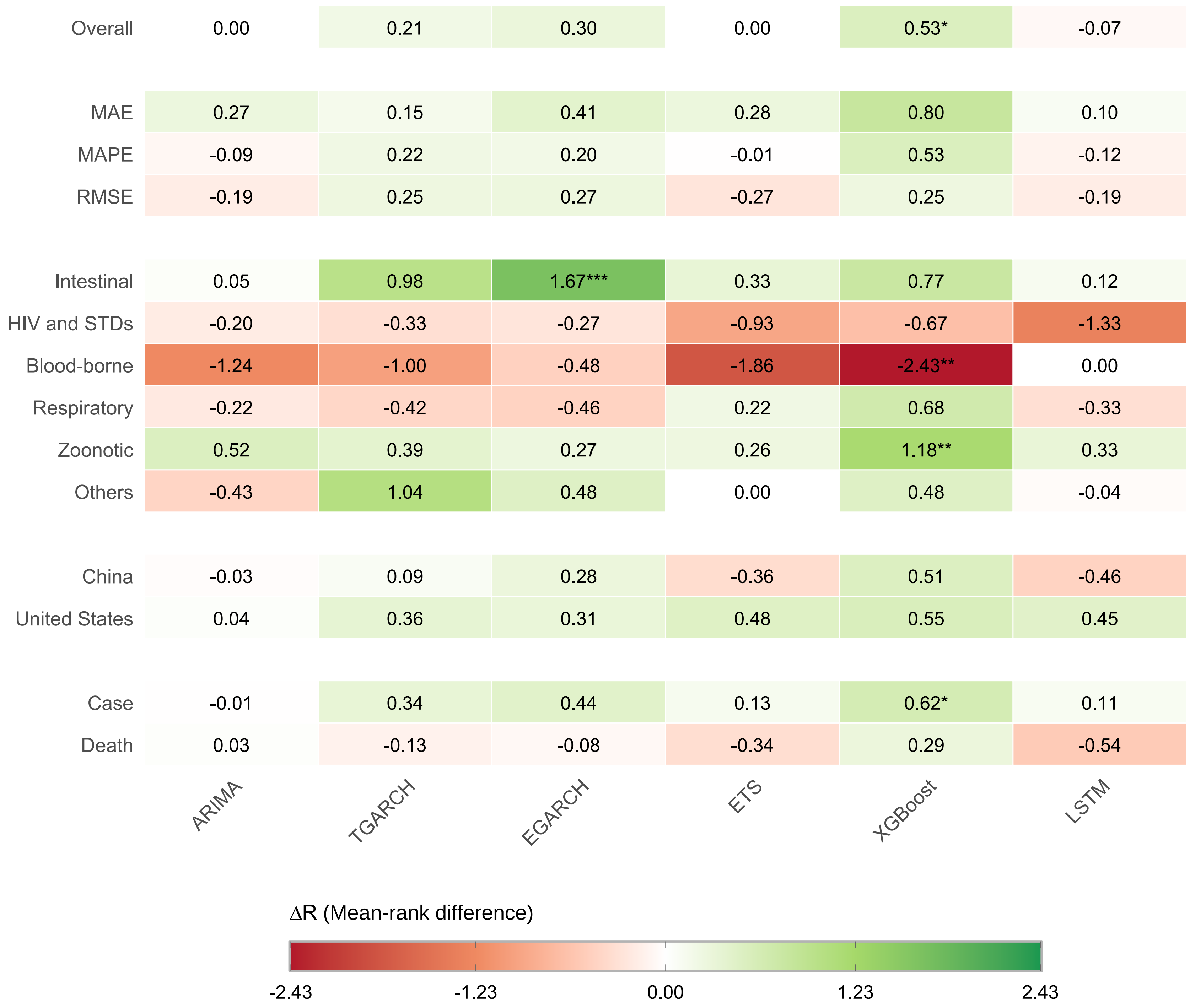
Overall
0.00
0.21
0.30
0.00
0.53*
-0.07
MAE
0.27
0.15
0.41
0.28
0.80
0.10
MAPE
-0.09
0.22
0.20
-0.01
0.53
-0.12
RMSE
-0.19
0.25
0.27
-0.27
0.25
-0.19
Intestinal
0.05
0.98
1.67***
0.33
0.77
0.12
HIV and STDs
-0.20
-0.33
-0.27
-0.93
-0.67
-1.33
Blood-borne
-1.24
-1.00
-0.48
-1.86
-2.43**
0.00
Respiratory
-0.22
-0.42
-0.46
0.22
0.68
-0.33
Zoonotic
0.52
0.39
0.27
0.26
1.18**
0.33
Others
-0.43
1.04
0.48
0.00
0.48
-0.04
China
-0.03
0.09
0.28
-0.36
0.51
-0.46
United States
0.04
0.36
0.31
0.48
0.55
0.45
Case
-0.01
0.34
0.44
0.13
0.62*
0.11
Death
0.03
-0.13
-0.08
-0.34
0.29
-0.54
ARIMA
TGARCH
EGARCH
ETS
XGBoost
LSTM
ΔR (Mean-rank difference)
-2.43
-1.23
0.00
1.23
2.43
