# Supplementary figures and images for: Large language models as versatile predictive engines for notifiable infectious diseases
Source: PLOS Digit Health. 2026 Jul 8;5(7):e0001527. doi: 10.1371/journal.pdig.0001527 (PMC13345230; doi:10.1371/journal.pdig.0001527)

**A**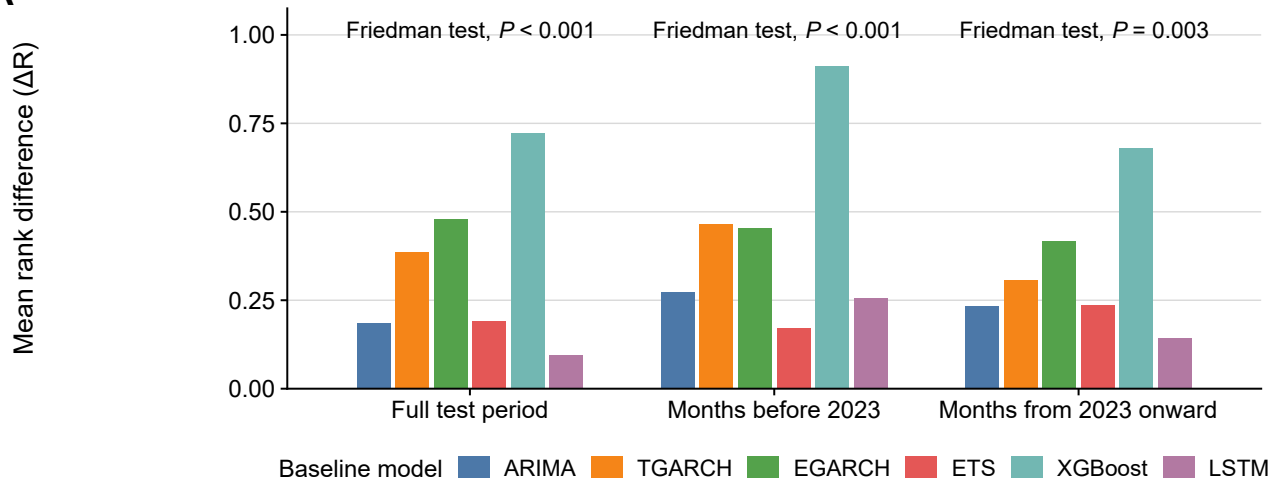**B**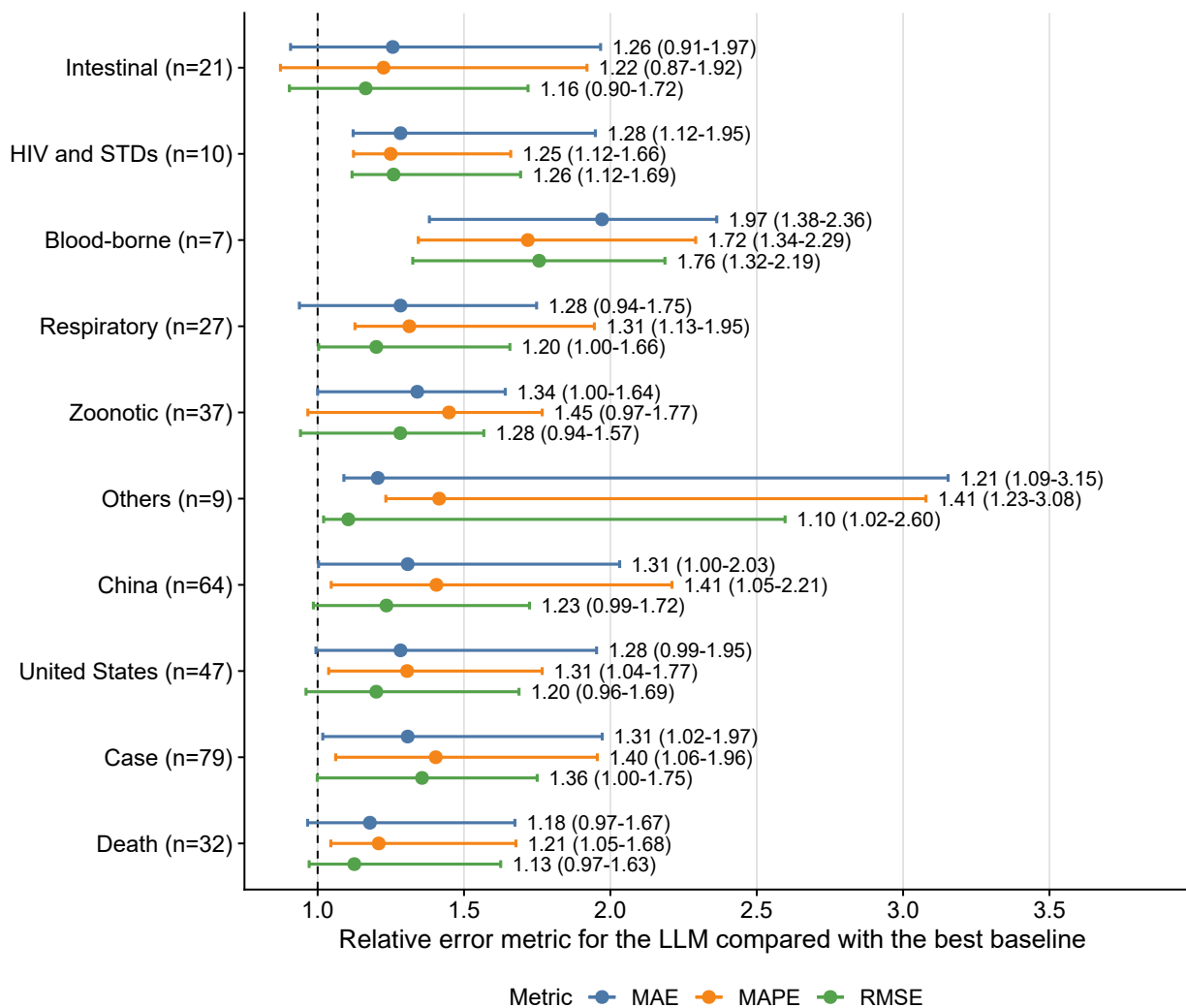

Supplement: S2 Fig — (A) Mean-rank differences (ΔR) for baseline models versus the LLM across the full test period, months before 2023, and months from 2023 onward. Positive values indicate better performance of the LLM. P values are from Friedman tests using the same framework as the primary analysis. (B) Relative MAE, relative MAPE, and relative RMSE for the LLM compared with the best baseline model by disease category, country, and outcome. Each relative error metric was calculated as the LLM error metric divided by the lowest corresponding error metric achieved by any baseline model for the same task. Points show medians, horizontal lines show interquartile ranges, and colors indicate MAE, MAPE, and RMSE. LLM, large language model–based regression; ARIMA, autoregressive integrated moving average; TGARCH, threshold generalized autoregressive conditional heteroskedasticity; EGARCH, exponential generalized autoregressive conditional heteroskedasticity; ETS, exponential smoothing state-space model; XGBoost, Extreme Gradient Boosting; LSTM, long short-term memory network; MAE, mean absolute error; MAPE, mean absolute percentage error; RMSE, root mean squared error. (PDF) [file pdig.0001527.s002.pdf]
