# Supplementary material for: Large language models as versatile predictive engines for notifiable infectious diseases
Source: PLOS Digit Health. 2026 Jul 8;5(7):e0001527. doi: 10.1371/journal.pdig.0001527 (PMC13345230; doi:10.1371/journal.pdig.0001527)
Supplement: S1 Table — (DOCX) [file pdig.0001527.s003.docx]

# S1 Table Excluded notifiable infectious diseases and corresponding exclusion reasons

| **Country** | **Disease** | **Reason** |
| --- | --- | --- |
| China | Lymphatic filariasis | 1 |
| China | Diphtheria | 1 |
| China | Poliomyelitis | 1 |
| China | Human infection with highly pathogenic avian influenza | 1 |
| China | Infectious atypical pneumonia | 1 |
| China | Plague | 1 |
| China | Cholera | 1 |
| China | COVID-19 | 2 |
| China | Mpox | 2 |
| China | Hepatitis D | 2 |
| China | Other hepatitis | 3 |
| China | Viral hepatitis (composite category) | 3 |
| China | Hand-foot-mouth disease | 4 |
| China | Pertussis | 4 |
| China | H7N9 avian influenza | 5 |
| United States | Anthrax | 1 |
| United States | Candidaauris,screening | 1 |
| United States | Carbapenemase-Producing Organisms,Total | 1 |
| United States | Carbapenemase-producing carbapenem-resistant Enterobacteriaceae | 1 |
| United States | Chancroid | 1 |
| United States | Cholera | 1 |
| United States | Coronavirus Disease 2019 (COVID-19), Total | 2 |
| United States | Dengue virus infections, Dengue-like illness | 1 |
| United States | Dengue virus infections, Severe dengue | 1 |
| United States | Diphtheria | 1 |
| United States | Ehrlichiosis and Anaplasmosis, Ehrlichia ewingii infection | 1 |
| United States | Hantavirus infection, non-hantavirus pulmonary syndrome | 1 |
| United States | Hantavirus pulmonary syndrome | 1 |
| United States | Hepatitis, B, perinatal infection | 1 |
| United States | Melioidosis | 1 |
| United States | Meningococcal disease, Other serogroups | 1 |
| United States | Meningococcal disease, Serogroup B | 1 |
| United States | Mpox | 2 |
| United States | Novel Influenza A virus infections | 1 |
| United States | Paratyphoid fever | 1 |
| United States | Pertussis | 4 |
| United States | Plague | 1 |
| United States | Poliomyelitis, paralytic | 1 |
| United States | Poliovirus infection, nonparalytic | 1 |
| United States | Psittacosis | 1 |
| United States | Rabies, Animal | 6 |
| United States | Rabies, Human | 1 |
| United States | Rubella | 1 |
| United States | Rubella, congenital syndrome | 1 |
| United States | Salmonellosis | 1 |
| United States | Salmonellosis (excluding paratyphoid fever and typhoid fever) | 1 |
| United States | Severe acute respiratory syndrome-associated coronavirus disease | 1 |
| United States | Smallpox | 1 |
| United States | Tetanus | 1 |
| United States | Toxic shock syndrome (other than Streptococcal) | 1 |
| United States | Trichinellosis | 1 |
| United States | Typhoid fever | 1 |
| United States | Vancomycin-resistant Staphylococcus aureus | 1 |
| United States | Viral hemorrhagic fevers | 1 |
| United States | Viral hemorrhagic fevers, Chapare virus | 1 |
| United States | Viral hemorrhagic fevers, Crimean-Congo hemorrhagic fever virus | 1 |
| United States | Viral hemorrhagic fevers, Ebola virus | 1 |
| United States | Viral hemorrhagic fevers, Guanarito virus | 1 |
| United States | Viral hemorrhagic fevers, Junin virus | 1 |
| United States | Viral hemorrhagic fevers, Lassa virus | 1 |
| United States | Viral hemorrhagic fevers, Lujo virus | 1 |
| United States | Viral hemorrhagic fevers, Machupo virus | 1 |
| United States | Viral hemorrhagic fevers, Marburg virus | 1 |
| United States | Viral hemorrhagic fevers, Sabia virus | 1 |
| United States | Yellow fever | 1 |
| United States | Zika virus, Zika virus disease, congenital | 1 |
| United States | Zika virus, Zika virus disease, non-congenital | 2 |
| United States | Zika virus, Zika virus infection, congenital | 1 |
| United States | Zika virus, Zika virus infection, non-congenital | 2 |

Criterion 1. Very low reported case counts with long stretches of zeros, leading to unstable estimation. Criterion 2. Recently emerged disease or short surveillance series with insufficient historical data. Criterion 3: Substantial changes in diagnostic or classification practices over time; temporal trends mainly reflect diagnostic rather than epidemiological changes. Criterion 4: Extreme temporal variability with frequent outbreaks or outliers that impair stable parameter estimation and model convergence. Criterion 5: Extremely low endemicity or near-elimination in the study setting. Criterion 6. Animal-only or non-human infection category (e.g., Rabies, Animal), not modeled as a human infectious disease outcome.
