# Supplementary material for: Large language models as versatile predictive engines for notifiable infectious diseases
Source: PLOS Digit Health. 2026 Jul 8;5(7):e0001527. doi: 10.1371/journal.pdig.0001527 (PMC13345230; doi:10.1371/journal.pdig.0001527)
Supplement: S2 Table — (DOCX) [file pdig.0001527.s004.docx]

# S2 Table Case and death numbers for 32 notifiable infectious diseases in China from January 2009 to February 2025.

| **Disease** | **Transmission Category** | **Cases Median (IQR)** | **Maximum Cases** | **Month of Maximum Cases** | **Deaths Median (IQR)** | **Maximum Deaths** | **Month of Maximum Deaths** |
| --- | --- | --- | --- | --- | --- | --- | --- |
| Bacterial and Amoebic Dysentery | Intestinal | 7896 (4358–14146) | 113322 | 2010-03 | 0 (0–1) | 8 | 2009-07 |
| Hepatitis A | Intestinal | 1722 (1233–2202) | 4399 | 2009-06 | 0 (0–1) | 4 | 2015-12 |
| Hepatitis E | Intestinal | 2265 (1944–2670) | 4262 | 2011-03 | 1 (1–2) | 6 | 2010-02 |
| Other Infectious Diarrheal Diseases | Intestinal | 89298 (67898–108357) | 242791 | 2025-02 | 2 (0–3) | 7 | 2010-11 |
| Typhoid and Paratyphoid Fever | Intestinal | 847 (583–1164) | 2164 | 2009-08 | 0 (0–0) | 3 | 2017-06 |
| AIDS | HIV and STDs | 4466 (3330–5322) | 7897 | 2018-12 | 1232 (962–1657) | 2486 | 2021-12 |
| Gonorrhea | HIV and STDs | 9122 (8194–10544) | 13803 | 2017-08 | 0 (0–0) | 4 | 2020-01 |
| Syphilis | HIV and STDs | 40071 (35152–46951) | 64161 | 2024-03 | 6 (4–8) | 25 | 2020-01 |
| Hepatitis B | Blood-borne | 100891 (93289–109930) | 152967 | 2024-03 | 40 (31–50) | 98 | 2009-12 |
| Hepatitis C | Blood-borne | 19070 (16064–20682) | 24666 | 2018-03 | 11 (8–16) | 589 | 2024-12 |
| Influenza | Respiratory | 21536 (10644–77045) | 4113326 | 2023-12 | 1 (0–4) | 455 | 2009-12 |
| Leprosy | Respiratory | 53 (35–76) | 209 | 2013-01 | 0 (0–0) | 1 | 2010-01 |
| Measles | Respiratory | 434 (113–1494) | 14371 | 2009-04 | 0 (0–1) | 12 | 2010-05 |
| Meningococcal Meningitis | Respiratory | 11 (6–18) | 138 | 2009-02 | 1 (0–2) | 22 | 2009-02 |
| Mumps | Respiratory | 15441 (10678–24800) | 71606 | 2012-06 | 0 (0–0) | 4 | 2020-01 |
| Rubella | Respiratory | 411 (109–1298) | 19173 | 2009-04 | 0 (0–0) | 1 | 2010-05 |
| Scarlet Fever | Respiratory | 3050 (1799–5382) | 13053 | 2019-12 | 0 (0–0) | 2 | 2013-12 |
| Tuberculosis | Respiratory | 92266 (72885–105745) | 142182 | 2009-06 | 169 (142–208) | 431 | 2009-07 |
| Anthrax | Zoonotic | 20 (15–35) | 123 | 2023-08 | 0 (0–0) | 2 | 2010-07 |
| Brucellosis | Zoonotic | 4254 (2694–5656) | 9943 | 2022-06 | 0 (0–0) | 2 | 2018-07 |
| Dengue Fever | Zoonotic | 28 (7–193) | 28796 | 2014-10 | 0 (0–0) | 4 | 2014-09 |
| Echinococcosis | Zoonotic | 339 (282–408) | 1077 | 2017-03 | 0 (0–0) | 5 | 2024-07 |
| Hemorrhagic Fever | Zoonotic | 722 (434–1021) | 3000 | 2012-11 | 4 (1–8) | 33 | 2010-12 |
| Japanese Encephalitis | Zoonotic | 14 (3–70) | 2066 | 2009-08 | 1 (0–5) | 92 | 2009-08 |
| Leishmaniasis | Zoonotic | 21 (16–27) | 123 | 2015-11 | 0 (0–0) | 1 | 2010-03 |
| Leptospirosis | Zoonotic | 19 (7–45) | 262 | 2010-09 | 0 (0–0) | 7 | 2010-10 |
| Malaria | Zoonotic | 236 (189–306) | 2511 | 2009-08 | 1 (0–2) | 5 | 2011-05 |
| Rabies | Zoonotic | 46 (17–106) | 238 | 2009-09 | 44 (15–95) | 260 | 2009-12 |
| Schistosomiasis | Zoonotic | 144 (6–426) | 10481 | 2015-11 | 0 (0–0) | 3 | 2012-06 |
| Typhus | Zoonotic | 131 (85–184) | 3318 | 2013-09 | 0 (0–0) | 1 | 2010-06 |
| Acute Hemorrhagic Conjunctivitis | Others | 2633 (2068–3324) | 225266 | 2010-09 | 0 (0–0) | 2 | 2020-02 |
| Neonatal Tetanus | Others | 11 (3–51) | 153 | 2009-08 | 0 (0–3) | 17 | 2009-12 |
