# Supplementary material for: Large language models as versatile predictive engines for notifiable infectious diseases
Source: PLOS Digit Health. 2026 Jul 8;5(7):e0001527. doi: 10.1371/journal.pdig.0001527 (PMC13345230; doi:10.1371/journal.pdig.0001527)
Supplement: S3 Table — (DOCX) [file pdig.0001527.s005.docx]

# S3 Table Case numbers for 47 notifiable infectious diseases in the United States from 2016 to 2023.

| **Disease** | **Transmission Category** | **Cases Median (IQR)** | **Maximum Cases** | **Month of Maximum Cases** |
| --- | --- | --- | --- | --- |
| Campylobacteriosis | Intestinal | 5232 (4439–6528) | 11403 | 2023-07 |
| Cryptosporidiosis, Total | Intestinal | 834 (652–1230) | 2541 | 2016-08 |
| Cyclosporiasis | Intestinal | 53 (14–211) | 2102 | 2023-07 |
| Giardiasis | Intestinal | 1131 (974–1374) | 2216 | 2016-12 |
| Hepatitis, A | Intestinal | 333 (163–828) | 2364 | 2018-12 |
| Listeriosis, Total | Intestinal | 72 (53–96) | 148 | 2021-07 |
| Salmonella Paratyphi infection | Intestinal | 3 (0–11) | 29 | 2022-12 |
| Salmonella Typhi infection | Intestinal | 14 (0–31) | 75 | 2023-08 |
| Salmonellosis (excluding S. Typhi infection and S. Paratyphi infection) | Intestinal | 2635 (0–4818) | 8799 | 2019-08 |
| Shiga toxin-producing Escherichia coli (STEC) | Intestinal | 1025 (734–1478) | 2695 | 2023-07 |
| Shigellosis | Intestinal | 1244 (980–1643) | 2887 | 2023-12 |
| Vibriosis, Total | Intestinal | 182 (111–285) | 658 | 2021-07 |
| Chlamydia trachomatis infection | HIV and STDs | 134431 (125580–157954) | 187239 | 2019-08 |
| Gonorrhea | HIV and STDs | 50242 (44520–56263) | 76904 | 2020-10 |
| Human immunodeficiency virus diagnoses | HIV and STDs | 3024 (2579–3242) | 3672 | 2022-08 |
| Syphilis, Total, all stages | HIV and STDs | 10894 (8773–15446) | 20795 | 2023-04 |
| Hepatitis, B, acute | Blood-borne | 224 (172–264) | 424 | 2016-12 |
| Hepatitis, C, acute | Blood-borne | 427 (348–495) | 758 | 2020-02 |
| Hepatitis, C, perinatal infection | Blood-borne | 15 (5–20) | 35 | 2019-03 |
| Coccidioidomycosis | Respiratory | 1364 (1186–1703) | 3671 | 2023-12 |
| Haemophilus influenzae, invasive disease, All ages, all serotypes | Respiratory | 416 (319–533) | 998 | 2023-12 |
| Invasive pneumococcal disease, All ages | Respiratory | 1330 (876–1966) | 3904 | 2023-12 |
| Legionellosis | Respiratory | 574 (384–858) | 1707 | 2021-07 |
| Measles, Total | Respiratory | 4 (0–19) | 299 | 2019-04 |
| Meningococcal disease, All serogroups | Respiratory | 26 (20–34) | 75 | 2023-12 |
| Meningococcal disease, Serogroups ACWY | Respiratory | 10 (7–14) | 42 | 2023-12 |
| Meningococcal disease, Unknown serogroup | Respiratory | 8 (6–12) | 33 | 2018-12 |
| Mumps | Respiratory | 121 (26–315) | 1975 | 2016-12 |
| Tuberculosis | Respiratory | 712 (602–800) | 1324 | 2016-12 |
| Varicella morbidity | Respiratory | 533 (339–724) | 1141 | 2023-12 |
| Arboviral diseases, Chikungunya virus disease | Zoonotic | 9 (4–17) | 34 | 2016-08 |
| Babesiosis, Total | Zoonotic | 87 (18–235) | 1335 | 2023-07 |
| Brucellosis | Zoonotic | 11 (8–14) | 26 | 2017-07 |
| Dengue virus infections, Dengue | Zoonotic | 44 (20–70) | 353 | 2023-09 |
| Ehrlichiosis and Anaplasmosis, Anaplasma phagocytophilum infection | Zoonotic | 274 (83–529) | 2543 | 2023-07 |
| Ehrlichiosis and Anaplasmosis, Ehrlichia chaffeensis infection | Zoonotic | 86 (24–198) | 576 | 2023-07 |
| Ehrlichiosis and Anaplasmosis, Undetermined ehrlichiosis/anaplasmosis | Zoonotic | 8 (2–17) | 78 | 2018-06 |
| Leptospirosis | Zoonotic | 6 (4–8) | 24 | 2018-09 |
| Lyme disease, Total | Zoonotic | 2414 (1193–4590) | 22233 | 2023-07 |
| Malaria | Zoonotic | 135 (95–204) | 365 | 2023-09 |
| Q fever, Total | Zoonotic | 15 (11–20) | 32 | 2018-03 |
| Spotted fever rickettsiosis, Total | Zoonotic | 153 (60–361) | 1364 | 2017-07 |
| Tularemia | Zoonotic | 17 (6–24) | 59 | 2019-06 |
| Botulism, Total | Others | 17 (13–20) | 37 | 2016-06 |
| Candida auris, clinical | Others | 16 (0–66) | 435 | 2023-12 |
| Hansen's disease | Others | 6 (4–8) | 19 | 2018-08 |
| Vancomycin-intermediate Staphylococcus aureus | Others | 6 (4–9) | 16 | 2017-09 |
