# Supplementary material for: Large language models as versatile predictive engines for notifiable infectious diseases
Source: PLOS Digit Health. 2026 Jul 8;5(7):e0001527. doi: 10.1371/journal.pdig.0001527 (PMC13345230; doi:10.1371/journal.pdig.0001527)
Supplement: S4 Table — (DOCX) [file pdig.0001527.s006.docx]

# S4 Table Global tests comparing model performance ranks.

| **Stratum** | **Friedman χ²** | ***P* value** |
| --- | --- | --- |
| Overall | 26.24 | <0.001 |
| MAE | 11.66 | 0.070 |
| MAPE | 8.31 | 0.216 |
| RMSE | 10.98 | 0.089 |
| Intestinal | 38.03 | <0.001 |
| HIV and STDs | 8.60 | 0.197 |
| Blood-borne | 25.20 | <0.001 |
| Respiratory | 17.99 | 0.006 |
| Zoonotic | 25.48 | <0.001 |
| Others | 6.13 | 0.409 |
| China | 30.57 | <0.001 |
| United States | 9.04 | 0.171 |
| Case | 20.72 | 0.002 |
| Death | 9.30 | 0.158 |

MAE, mean absolute error; MAPE, mean absolute percentage error; RMSE, root mean squared error.
