# Supplementary material for: Large language models as versatile predictive engines for notifiable infectious diseases
Source: PLOS Digit Health. 2026 Jul 8;5(7):e0001527. doi: 10.1371/journal.pdig.0001527 (PMC13345230; doi:10.1371/journal.pdig.0001527)
Supplement: S5 Table — (DOCX) [file pdig.0001527.s007.docx]

# S5 Table Component-level attention weights by transmission category.

| **Transmission category** | **Date, %** | **Disease, %** | **Outcome, %** |
| --- | --- | --- | --- |
| Intestinal | 41.9 [39.0–44.7] | 22.6 [20.9–27.9] | 34.1 [32.1–36.1] |
| HIV and STDs | 47.9 [43.2–51.5] | 14.5 [10.6–24.6] | 35.3 [32.8–36.8] |
| Blood-borne | 46.3 [42.4–57.5] | 16.3 [15.7–22.1] | 33.1 [26.7–36.3] |
| Respiratory | 43.4 [39.9–48.2] | 18.4 [15.9–23.0] | 35.9 [32.3–38.2] |
| Zoonotic | 42.1 [38.4–48.5] | 20.8 [18.0–24.1] | 35.1 [32.4–37.3] |
| Others | 40.3 [38.8–41.9] | 24.2 [21.6–25.5] | 35.6 [33.3–37.9] |
