# Supplementary material for: Large language models as versatile predictive engines for notifiable infectious diseases
Source: PLOS Digit Health. 2026 Jul 8;5(7):e0001527. doi: 10.1371/journal.pdig.0001527 (PMC13345230; doi:10.1371/journal.pdig.0001527)
Supplement: S6 Table — (DOCX) [file pdig.0001527.s008.docx]

# S6 Table Global tests comparing model performance ranks (ablation without temporal embeddings).

| **Stratum** | **Friedman χ²** | ***P* value** |
| --- | --- | --- |
| Overall | 19.81 | 0.003 |
| MAE | 9.91 | 0.129 |
| MAPE | 7.16 | 0.306 |
| RMSE | 8.15 | 0.227 |
| Intestinal | 32.30 | <0.001 |
| HIV and STDs | 8.51 | 0.203 |
| Blood-borne | 22.98 | <0.001 |
| Respiratory | 16.98 | 0.009 |
| Zoonotic | 19.39 | 0.004 |
| Others | 6.93 | 0.327 |
| China | 27.68 | <0.001 |
| United States | 8.39 | 0.211 |
| Case | 17.36 | 0.008 |
| Death | 8.43 | 0.208 |

MAE, mean absolute error; MAPE, mean absolute percentage error; RMSE, root mean squared error.
