# Supplementary material for: Large language models as versatile predictive engines for notifiable infectious diseases
Source: PLOS Digit Health. 2026 Jul 8;5(7):e0001527. doi: 10.1371/journal.pdig.0001527 (PMC13345230; doi:10.1371/journal.pdig.0001527)
Supplement: S7 Table — (DOCX) [file pdig.0001527.s009.docx]

# S7 Table Post-hoc comparisons for baseline models versus the LLM using mean rank differences (ΔR) (ablation without temporal embeddings).

| **Stratum** | **ARIMA** | | **TGARCH** | | **EGARCH** | | **ETS** | | **XGBoost** | | **LSTM** | |
| --- | --- | --- | --- | --- | --- | --- | --- | --- | --- | --- | --- | --- |
|  | **ΔR** | ***P* value** | **ΔR** | ***P* value** | **ΔR** | ***P* value** | **ΔR** | ***P* value** | **ΔR** | ***P* value** | **ΔR** | ***P* value** |
| Overall | 0.00 | 1.000 | 0.21 | 0.882 | 0.30 | 0.584 | 0.00 | 1.000 | 0.53 | 0.029 | -0.07 | 1.000 |
| MAE | 0.27 | 0.965 | 0.15 | 0.998 | 0.41 | 0.795 | 0.28 | 0.962 | 0.80 | 0.083 | 0.10 | 1.000 |
| MAPE | -0.09 | 1.000 | 0.22 | 0.990 | 0.20 | 0.994 | -0.01 | 1.000 | 0.53 | 0.556 | -0.12 | 1.000 |
| RMSE | -0.19 | 0.995 | 0.25 | 0.977 | 0.27 | 0.967 | -0.27 | 0.967 | 0.25 | 0.977 | -0.19 | 0.995 |
| Intestinal | 0.05 | 1.000 | 0.98 | 0.126 | 1.67 | <0.001 | 0.33 | 0.975 | 0.77 | 0.380 | 0.12 | 1.000 |
| HIV and STDs | -0.20 | 1.000 | -0.33 | 0.997 | -0.27 | 0.999 | -0.93 | 0.634 | -0.67 | 0.896 | -1.33 | 0.202 |
| Blood-borne | -1.24 | 0.509 | -1.00 | 0.745 | -0.48 | 0.992 | -1.86 | 0.078 | -2.43 | 0.005 | 0.00 | 1.000 |
| Respiratory | -0.22 | 0.995 | -0.42 | 0.893 | -0.46 | 0.836 | 0.22 | 0.996 | 0.68 | 0.438 | -0.33 | 0.962 |
| Zoonotic | 0.52 | 0.557 | 0.39 | 0.829 | 0.27 | 0.971 | 0.26 | 0.976 | 1.18 | 0.001 | 0.33 | 0.919 |
| Others | -0.43 | 0.994 | 1.04 | 0.657 | 0.48 | 0.989 | 0.00 | 1.000 | 0.48 | 0.989 | -0.04 | 1.000 |
| China | -0.03 | 1.000 | 0.09 | 1.000 | 0.28 | 0.865 | -0.36 | 0.677 | 0.51 | 0.254 | -0.46 | 0.374 |
| United States | 0.04 | 1.000 | 0.36 | 0.799 | 0.31 | 0.895 | 0.48 | 0.516 | 0.55 | 0.323 | 0.45 | 0.591 |
| Case | -0.01 | 1.000 | 0.34 | 0.615 | 0.44 | 0.295 | 0.13 | 0.995 | 0.62 | 0.029 | 0.11 | 0.998 |
| Death | 0.03 | 1.000 | -0.13 | 1.000 | -0.08 | 1.000 | -0.34 | 0.937 | 0.29 | 0.973 | -0.54 | 0.622 |

ΔR, mean-rank difference. LLM, large language model–based regression; ARIMA, autoregressive integrated moving average; TGARCH, threshold generalized autoregressive conditional heteroskedasticity; EGARCH, exponential generalized autoregressive conditional heteroskedasticity; ETS, exponential smoothing state-space model; XGBoost, Extreme Gradient Boosting; LSTM, long short-term memory network; MAE, mean absolute error; MAPE, mean absolute percentage error; RMSE, root mean squared error.
