# Supplementary material for: Large language models as versatile predictive engines for notifiable infectious diseases
Source: PLOS Digit Health. 2026 Jul 8;5(7):e0001527. doi: 10.1371/journal.pdig.0001527 (PMC13345230; doi:10.1371/journal.pdig.0001527)
Supplement: S8 Table — (DOCX) [file pdig.0001527.s010.docx]

# S8 Table Zero-shot prediction performance on the test set (pretraining-contamination sensitivity analysis).

| **Country** | **Disease** | **Task** | **MAE** | **MAPE** | **RMSE** |
| --- | --- | --- | --- | --- | --- |
| CHINA | Bacterial and Amoebic Dysentery | Cases | 1.01E+18 | 5.96E+16 | 4.52E+18 |
| CHINA | Bacterial and Amoebic Dysentery | Deaths | 1.01E+18 | 5.06E+27 | 4.52E+18 |
| CHINA | Hepatitis A | Cases | 1.36E+05 | 1.42E+04 | 1.65E+05 |
| CHINA | Hepatitis A | Deaths | 1.37E+05 | 1.26E+15 | 1.66E+05 |
| CHINA | Hepatitis E | Cases | 1.23E+08 | 6.07E+06 | 3.90E+08 |
| CHINA | Hepatitis E | Deaths | 1.23E+08 | 3.09E+17 | 3.90E+08 |
| CHINA | Other Infectious Diarrheal Diseases | Cases | 1.13E+05 | 1.33E+02 | 1.17E+05 |
| CHINA | Other Infectious Diarrheal Diseases | Deaths | 1.31E+05 | 1.01E+15 | 1.63E+05 |
| CHINA | Typhoid and Paratyphoid Fever | Cases | 5.05E+17 | 1.04E+17 | 3.20E+18 |
| CHINA | Typhoid and Paratyphoid Fever | Deaths | 5.05E+17 | 5.05E+27 | 3.20E+18 |
| CHINA | AIDS | Cases | 1.69E+05 | 3.90E+03 | 1.82E+05 |
| CHINA | AIDS | Deaths | 1.71E+05 | 1.00E+04 | 1.85E+05 |
| CHINA | Gonorrhea | Cases | 1.57E+05 | 1.88E+03 | 1.73E+05 |
| CHINA | Gonorrhea | Deaths | 1.62E+05 | 1.37E+15 | 1.81E+05 |
| CHINA | Syphilis | Cases | 1.35E+05 | 2.95E+02 | 1.41E+05 |
| CHINA | Syphilis | Deaths | 1.62E+05 | 1.26E+09 | 1.81E+05 |
| CHINA | Hepatitis B | Cases | 9.96E+04 | 8.87E+01 | 1.03E+05 |
| CHINA | Hepatitis B | Deaths | 1.37E+05 | 4.84E+05 | 1.66E+05 |
| CHINA | Hepatitis C | Cases | 1.31E+05 | 7.49E+02 | 1.52E+05 |
| CHINA | Hepatitis C | Deaths | 1.37E+05 | 4.65E+05 | 1.66E+05 |
| CHINA | Influenza | Cases | 1.13E+06 | 2.76E+02 | 3.36E+06 |
| CHINA | Influenza | Deaths | 6.52E+05 | 6.07E+14 | 3.20E+06 |
| CHINA | Leprosy | Cases | 6.52E+05 | 2.75E+06 | 3.20E+06 |
| CHINA | Leprosy | Deaths | 6.52E+05 | 6.47E+15 | 3.20E+06 |
| CHINA | Measles | Cases | 1.62E+05 | 2.21E+05 | 1.81E+05 |
| CHINA | Measles | Deaths | 1.62E+05 | 1.62E+15 | 1.81E+05 |
| CHINA | Meningococcal Meningitis | Cases | 5.05E+17 | 5.05E+18 | 3.20E+18 |
| CHINA | Meningococcal Meningitis | Deaths | 5.05E+17 | 5.05E+27 | 3.20E+18 |
| CHINA | Mumps | Cases | 6.12E+05 | 7.94E+03 | 3.20E+06 |
| CHINA | Mumps | Deaths | 6.12E+05 | 6.12E+15 | 3.20E+06 |
| CHINA | Rubella | Cases | 1.62E+05 | 2.30E+05 | 1.81E+05 |
| CHINA | Rubella | Deaths | 1.62E+05 | 1.57E+15 | 1.81E+05 |
| CHINA | Scarlet Fever | Cases | 1.11E+05 | 6.96E+03 | 1.48E+05 |
| CHINA | Scarlet Fever | Deaths | 1.11E+05 | 1.11E+15 | 1.50E+05 |
| CHINA | Tuberculosis | Cases | 5.05E+17 | 8.18E+14 | 3.20E+18 |
| CHINA | Tuberculosis | Deaths | 5.05E+17 | 3.01E+17 | 3.20E+18 |
| CHINA | Anthrax | Cases | 6.18E+05 | 1.67E+06 | 3.20E+06 |
| CHINA | Anthrax | Deaths | 6.18E+05 | 6.02E+15 | 3.20E+06 |
| CHINA | Brucellosis | Cases | 5.05E+17 | 1.03E+16 | 3.20E+18 |
| CHINA | Brucellosis | Deaths | 5.05E+17 | 5.05E+27 | 3.20E+18 |
| CHINA | Dengue Fever | Cases | 1.11E+05 | 1.01E+14 | 1.49E+05 |
| CHINA | Dengue Fever | Deaths | 1.11E+05 | 1.11E+15 | 1.50E+05 |
| CHINA | Echinococcosis | Cases | 1.97E+05 | 7.10E+04 | 1.99E+05 |
| CHINA | Echinococcosis | Deaths | 1.97E+05 | 1.82E+15 | 2.00E+05 |
| CHINA | Hemorrhagic Fever | Cases | 5.05E+17 | 2.10E+16 | 3.20E+18 |
| CHINA | Hemorrhagic Fever | Deaths | 5.05E+17 | 1.94E+18 | 3.20E+18 |
| CHINA | Japanese Encephalitis | Cases | 6.43E+05 | 1.06E+14 | 3.20E+06 |
| CHINA | Japanese Encephalitis | Deaths | 6.43E+05 | 9.15E+14 | 3.20E+06 |
| CHINA | Leishmaniasis | Cases | 1.62E+05 | 7.99E+05 | 1.81E+05 |
| CHINA | Leishmaniasis | Deaths | 1.62E+05 | 1.42E+15 | 1.81E+05 |
| CHINA | Leptospirosis | Cases | 6.12E+05 | 1.87E+06 | 3.20E+06 |
| CHINA | Leptospirosis | Deaths | 6.12E+05 | 6.07E+15 | 3.20E+06 |
| CHINA | Malaria | Cases | 1.31E+05 | 1.36E+05 | 1.63E+05 |
| CHINA | Malaria | Deaths | 1.31E+05 | 5.56E+14 | 1.63E+05 |
| CHINA | Rabies | Cases | 6.52E+05 | 5.34E+06 | 3.20E+06 |
| CHINA | Rabies | Deaths | 6.52E+05 | 5.99E+06 | 3.20E+06 |
| CHINA | Schistosomiasis | Cases | 5.05E+17 | 1.26E+19 | 3.20E+18 |
| CHINA | Schistosomiasis | Deaths | 5.05E+17 | 5.05E+27 | 3.20E+18 |
| CHINA | Typhus | Cases | 6.93E+05 | 4.47E+05 | 3.22E+06 |
| CHINA | Typhus | Deaths | 6.93E+05 | 6.93E+15 | 3.22E+06 |
| CHINA | Acute Hemorrhagic Conjunctivitis | Cases | 5.05E+17 | 2.01E+16 | 3.20E+18 |
| CHINA | Acute Hemorrhagic Conjunctivitis | Deaths | 5.05E+17 | 5.05E+27 | 3.20E+18 |
| CHINA | Neonatal Tetanus | Cases | 5.05E+17 | 5.05E+19 | 3.20E+18 |
| CHINA | Neonatal Tetanus | Deaths | 5.05E+17 | 5.05E+27 | 3.20E+18 |
| US | Arboviral diseases, Chikungunya virus disease | Cases | 2.02E+05 | 3.17E+06 | 2.02E+05 |
| US | Babesiosis, Total | Cases | 4.04E+18 | 2.27E+18 | 9.04E+18 |
| US | Botulism, Total | Cases | 9.88E+08 | 5.46E+09 | 1.10E+09 |
| US | Brucellosis | Cases | 2.02E+18 | 1.80E+19 | 6.40E+18 |
| US | Campylobacteriosis | Cases | 8.08E+04 | 1.24E+03 | 1.24E+05 |
| US | Candida auris, clinical | Cases | 1.01E+18 | 9.63E+17 | 4.52E+18 |
| US | Chlamydia trachomatis infection | Cases | 1.01E+18 | 6.22E+14 | 4.52E+18 |
| US | Coccidioidomycosis | Cases | 2.42E+05 | 1.69E+04 | 4.82E+05 |
| US | Cryptosporidiosis, Total | Cases | 6.18E+07 | 6.00E+06 | 8.73E+07 |
| US | Cyclosporiasis | Cases | 2.63E+05 | 4.20E+05 | 4.87E+05 |
| US | Dengue virus infections, Dengue | Cases | 2.02E+18 | 2.06E+18 | 6.40E+18 |
| US | Ehrlichiosis and Anaplasmosis, Anaplasma phagocytophilum infection | Cases | 1.92E+05 | 1.13E+05 | 1.97E+05 |
| US | Ehrlichiosis and Anaplasmosis, Ehrlichia chaffeensis infection | Cases | 2.02E+05 | 4.03E+05 | 2.02E+05 |
| US | Ehrlichiosis and Anaplasmosis, Undetermined ehrlichiosis/anaplasmosis | Cases | 2.02E+05 | 1.01E+14 | 2.02E+05 |
| US | Giardiasis | Cases | 1.17E+06 | 8.03E+04 | 4.53E+06 |
| US | Gonorrhea | Cases | 4.05E+18 | 7.20E+15 | 9.05E+18 |
| US | Haemophilus influenzae, invasive disease, All ages, all serotypes | Cases | 9.26E+08 | 1.97E+08 | 1.07E+09 |
| US | Hansen's disease | Cases | 1.26E+06 | 1.54E+07 | 4.55E+06 |
| US | Hepatitis, A | Cases | 2.02E+18 | 1.47E+18 | 6.40E+18 |
| US | Hepatitis, B, acute | Cases | 1.11E+19 | 6.41E+18 | 1.50E+19 |
| US | Hepatitis, C, acute | Cases | 7.08E+18 | 1.57E+18 | 1.20E+19 |
| US | Hepatitis, C, perinatal infection | Cases | 5.21E+04 | 2.93E+05 | 1.01E+05 |
| US | Human immunodeficiency virus diagnoses | Cases | 9.26E+08 | 2.82E+07 | 1.07E+09 |
| US | Invasive pneumococcal disease, All ages | Cases | 1.82E+05 | 1.40E+04 | 4.69E+05 |
| US | Legionellosis | Cases | 1.01E+18 | 1.17E+17 | 4.52E+18 |
| US | Leptospirosis | Cases | 1.27E+06 | 1.33E+07 | 4.55E+06 |
| US | Listeriosis, Total | Cases | 1.50E+18 | 1.89E+18 | 3.87E+18 |
| US | Lyme disease, Total | Cases | 3.03E+18 | 5.79E+16 | 7.83E+18 |
| US | Malaria | Cases | 1.24E+08 | 8.45E+07 | 3.90E+08 |
| US | Measles, Total | Cases | 5.04E+18 | 1.01E+28 | 9.58E+18 |
| US | Meningococcal disease, All serogroups | Cases | 3.03E+18 | 1.00E+19 | 7.83E+18 |
| US | Meningococcal disease, Serogroups ACWY | Cases | 1.01E+18 | 3.75E+18 | 4.52E+18 |
| US | Meningococcal disease, Unknown serogroup | Cases | 1.42E+05 | 2.25E+06 | 1.69E+05 |
| US | Mumps | Cases | 2.02E+18 | 3.70E+18 | 6.40E+18 |
| US | Q fever, Total | Cases | 1.62E+19 | 1.09E+20 | 1.81E+19 |
| US | Salmonella Paratyphi infection | Cases | 1.01E+18 | 9.19E+18 | 4.52E+18 |
| US | Salmonella Typhi infection | Cases | 1.01E+18 | 3.26E+18 | 4.52E+18 |
| US | Salmonellosis (excluding S. Typhi infection and S. Paratyphi infection) | Cases | 1.17E+06 | 2.03E+04 | 4.53E+06 |
| US | Shiga toxin-producing Escherichia coli (STEC) | Cases | 2.01E+05 | 1.39E+04 | 2.01E+05 |
| US | Shigellosis | Cases | 7.08E+18 | 4.62E+17 | 1.20E+19 |
| US | Spotted fever rickettsiosis, Total | Cases | 2.51E+18 | 2.77E+18 | 5.95E+18 |
| US | Syphilis, Total, all stages | Cases | 2.02E+18 | 1.13E+16 | 6.40E+18 |
| US | Tuberculosis | Cases | 1.24E+08 | 1.82E+07 | 3.90E+08 |
| US | Tularemia | Cases | 2.43E+05 | 2.62E+06 | 4.83E+05 |
| US | Vancomycin-intermediate Staphylococcus aureus | Cases | 1.01E+18 | 1.01E+20 | 4.52E+18 |
| US | Varicella morbidity | Cases | 6.17E+08 | 1.50E+08 | 8.73E+08 |
| US | Vibriosis, Total | Cases | 7.08E+18 | 2.29E+18 | 1.20E+19 |
