# Supplementary material for: Large language models as versatile predictive engines for notifiable infectious diseases
Source: PLOS Digit Health. 2026 Jul 8;5(7):e0001527. doi: 10.1371/journal.pdig.0001527 (PMC13345230; doi:10.1371/journal.pdig.0001527)
Supplement: S9 Table — (DOCX) [file pdig.0001527.s011.docx]

# S9 Table Sensitivity analysis by evaluation period using mean rank differences (ΔR).

| **Period** | **Friedman *P* value** | **ARIMA ΔR** | **TGARCH ΔR** | **EGARCH ΔR** | **ETS ΔR** | **XGBoost ΔR** | **LSTM ΔR** |
| --- | --- | --- | --- | --- | --- | --- | --- |
| Full test period | <0.001 | 0.19 | 0.39 | 0.48 | 0.19 | 0.72 | 0.09 |
| Months before 2023 | <0.001 | 0.27 | 0.47 | 0.45 | 0.17 | 0.91 | 0.26 |
| Months from 2023 onward | 0.003 | 0.23 | 0.31 | 0.42 | 0.24 | 0.68 | 0.14 |

ΔR, mean-rank difference. LLM, large language model–based regression; ARIMA, autoregressive integrated moving average; TGARCH, threshold generalized autoregressive conditional heteroskedasticity; EGARCH, exponential generalized autoregressive conditional heteroskedasticity; ETS, exponential smoothing state-space model; XGBoost, Extreme Gradient Boosting; LSTM, long short-term memory network.
