# Supplementary material for: Large language models as versatile predictive engines for notifiable infectious diseases
Source: PLOS Digit Health. 2026 Jul 8;5(7):e0001527. doi: 10.1371/journal.pdig.0001527 (PMC13345230; doi:10.1371/journal.pdig.0001527)
Supplement: S10 Table — (DOCX) [file pdig.0001527.s012.docx]

# S10 Table Data sources used in the study.

| **Source** | **Geographic scope** | **Study period** |
| --- | --- | --- |
| China National Notifiable Diseases Surveillance System, accessed through the China CDC | China | January 2009 to February 2025 |
| United States National Notifiable Diseases Surveillance System, accessed through CDC WONDER | United States | 2016 to 2023 |

CDC, Centers for Disease Control and Prevention. The data supporting the findings of this study are available online: <https://www.chinacdc.cn/jksj/jksj01/index.html> and <https://wonder.cdc.gov/nndss-annual-summary.html>
